# Environmental exposures in autoimmune inflammatory rheumatic diseases: a priority-based evidence map, immune-mechanism integration audit and research agenda

**DOI:** 10.3389/fimmu.2026.1916679

**Published:** 2026-08-10

**Authors:** Chuanwei Zhang, Lijun Pang, Ziheng Zhu, Jianing Wang, Chuanbing Huang

**Affiliations:** 1 Department of Rheumatology, The First Affiliated Hospital of Anhui University of Chinese Medicine, Hefei, China; 2 Center for Xin’an Medicine and Modernization of Traditional Chinese Medicine of IHM, Hefei, China

**Keywords:** autoimmune inflammatory rheumatic diseases, environmental epidemiology, evidence gap map, exposome, immune biomarkers, research priority, risk of bias

## Abstract

**Introduction:**

Environmental exposures have been hypothesised to influence immune tolerance, but human studies of autoimmune inflammatory rheumatic diseases (AIRDs) often report exposure–disease associations without measuring immune intermediates. We mapped the structure, directionality, immune-mechanism integration and research priorities of this literature.

**Methods:**

Web of Science Core Collection and Scopus were searched on 18 May 2026 for English-language article/review records published during 2005–2025; non-original publications were excluded during eligibility assessment. Of 12,497 deduplicated records screened at title/abstract level, a predefined prioritisation strategy selected 2,632 reports for focused coding. Four high-density exposure–AIRD cells underwent SWiM-aligned direction-of-effect synthesis and focused OHAT-style risk-of-bias assessment, 64 cells underwent consensus research-priority scoring, and a post hoc four-level audit assessed immune-mechanism integration. No pooled effect estimates were calculated because exposures, outcomes, designs and analytical approaches were heterogeneous.

**Results:**

Of 17,089 records before deduplication, 433 studies were included. Smoking/tobacco exposures dominated (n = 215, 49.7%), and rheumatoid arthritis was the most studied AIRD (n = 254, 58.7%). Twenty-five studies (5.8%) directly measured a pathway-proximal intermediary or omics readout, 79 (18.2%) measured an immune, serologic, complement or inflammatory biomarker, and 266 (61.4%) did not integrate an immune endpoint. Among 199 classifiable study-cell entries in four dense cells, mixed (n = 93) and positive/harmful (n = 91) directions were nearly balanced. Alternative priority-scoring schemes showed high rank concordance, and smoking/tobacco × systemic sclerosis remained within the top three priorities.

**Discussion:**

This priority-based evidence map identifies a large but uneven association literature with limited direct immune-mechanism integration and supports more rigorous exposure assessment, immune phenotyping, mixture-aware analyses and geographically diverse research.

## 1 Introduction

Autoimmune inflammatory rheumatic diseases (AIRDs), including rheumatoid arthritis, systemic lupus erythematosus, systemic sclerosis, Sjögren’s syndrome, idiopathic inflammatory myopathies, vasculitides and spondyloarthritis-related diseases, are understood to reflect interactions among genetic susceptibility, environmental context and dysregulated immune tolerance (1–4). Although these diseases are clinically heterogeneous, they share immune-mediated features such as persistent innate and adaptive immune activation, autoantibody formation, tissue inflammation and organ-specific damage. A cross-exposure view is therefore needed to determine how environmental factors have been evaluated in relation to AIRD occurrence, phenotype and immune features, and where evidence remains too sparse for disease-specific inference.

Historically, environmental AIRD research has centred on cigarette smoking in rheumatoid arthritis and silica or occupational dust exposure in systemic sclerosis and related autoimmune conditions (5–8). More recent work has expanded toward ambient air pollution, traffic-related exposures, heavy metals and trace elements, pesticides, solvents, persistent organic pollutants, per- and polyfluoroalkyl substances (PFAS), endocrine-disrupting chemicals and broader occupational or exposomic profiles (9–15). These exposures are immunologically relevant because they may converge on oxidative stress, epithelial barrier injury, mucosal immune activation, post-translational protein modification, epigenetic alteration, endocrine disruption and dysregulated innate or adaptive immune responses (14, 16–19).

Environmental exposures can be interpreted within hypotheses of mucosal or epithelial immune dysregulation rather than only as isolated epidemiological variables. Inhaled, dermal, occupational and dietary-contact exposures may be relevant at the lung, oral cavity, gut, skin or vascular endothelium. Experimental and clinical literature has proposed that tobacco smoke, silica, mineral dusts, particulate matter and traffic-related pollutants may be associated with barrier injury, altered antigen presentation, oxidative stress and innate immune activation. In rheumatoid arthritis, smoking and inhalational exposures have been studied in relation to peptidylarginine deiminase activity, protein citrullination, anti-citrullinated protein antibodies and interaction with HLA-DRB1 shared-epitope alleles. These pathways provide biologically plausible hypotheses linking exposure assessment, preclinical autoimmunity and clinically apparent inflammatory arthritis, but they require direct testing in human populations.

Several mapped exposure classes have also been discussed in relation to oxidative and inflammatory pathways. Ambient particulate matter, metals, organic solvents, pesticides and persistent organic pollutants have been associated in experimental or non-AIRD settings with reactive oxygen species, mitochondrial dysfunction, redox-sensitive transcription and altered cytokine networks. These observations provide hypotheses involving modified self-antigens, impaired clearance of damaged material, inflammasome signalling, neutrophil extracellular traps and type I interferon responses. Such pathways are relevant to systemic lupus erythematosus and vasculitis, but the human AIRD evidence directly linking measured exposures to these intermediates is substantially thinner than the literature reporting disease occurrence, activity or prevalence.

Endocrine-disrupting chemicals, PFAS, bisphenols, phthalates and other synthetic chemicals may be relevant through partly different immunological hypotheses, including nuclear-receptor signalling, sex-hormone pathways, epigenetic regulation, B-cell activation, T-cell differentiation and cytokine balance. These hypotheses are pertinent because many AIRDs show marked sex differences, fluctuating activity and prominent autoantibody phenotypes (20). Epigenetic measures, including DNA methylation, histone modification and microRNA regulation, may provide testable links between chronic exposure and immune phenotype. Disease-specific interpretation is also necessary: epithelial immune activation may be particularly relevant to Sjögren’s syndrome; endothelial, vascular and immune–fibrotic pathways to systemic sclerosis; and mucosal immunity and the IL-23/IL-17 axis to spondyloarthritis-spectrum disease. These mechanisms were treated as interpretive hypotheses, not as demonstrated consequences of the mapped exposures.

These mechanistic considerations guided interpretation but were not treated as evidence of causality. Many mapped studies reported disease occurrence, prevalence, activity, serology or clinical phenotype without directly measuring immune intermediates, whereas many basic or non-AIRD mechanistic studies were ineligible because they lacked a human exposure–AIRD link. The present synthesis therefore uses biological plausibility to structure interpretation and research-priority scoring, not to infer that an exposure causes an AIRD. To make this distinction measurable, the revision adds a *post hoc* audit of whether included studies directly assessed immune biomarkers, pathway-proximal intermediates, omics readouts, immune-defined phenotypes or genetic susceptibility within the exposure analysis.

Existing syntheses of environmental exposures and rheumatic diseases have often focused on single exposures, single diseases or narrow mechanistic questions (5, 8, 10, 12, 15). This approach is useful for evaluating specific hypotheses, but it does not reveal how the environmental-immunology literature as a whole is structured (21–23). In particular, it remains unclear which exposure-AIRD combinations are well represented, which are supported by only sparse evidence, and which remain essentially unstudied despite plausible immune mechanisms or public-health relevance (21, 22).

An evidence map can address this gap by characterising the volume and distribution of evidence rather than attempting to pool heterogeneous effect estimates (21, 22, 24). Evidence mapping is particularly suitable when a field is broad, methodologically diverse and unevenly developed (21, 22, 25). For environmental immunology and AIRD research, such a map can help distinguish mature exposure-disease areas from underdeveloped immunologically plausible spaces, identify opportunities for future exposure-science and autoimmune-disease studies, and provide a transparent foundation for targeted systematic reviews (21, 22, 26).

We therefore conducted a priority-based evidence map informed by systematic searching and dual title/abstract screening to characterise environmental exposure research across AIRDs (21, 22). Using Web of Science Core Collection and Scopus, we screened a large deduplicated corpus and constructed a focused priority-coded subset. Structured additions included dense-cell direction-of-effect synthesis with focused risk-of-bias contextualisation, consensus research-priority scoring, a *post hoc* audit of immune-mechanism integration, and robustness analyses of the priority ranking. Our objectives were to identify which exposure–AIRD combinations are well represented, where evidence is sparse, how directional patterns differ across dense cells, how often studies directly integrate immune endpoints, and which cells warrant targeted future research.

## 2 Materials and methods

### 2.1 Study design and reporting approach

We conducted a priority-based mapping review to characterise human studies of environmental exposures and AIRDs. The primary objective was to map evidence volume and structure across exposure domains, AIRD categories, geographic settings, study designs and methodological features rather than to estimate a single pooled exposure–outcome effect. Secondary structured layers comprised dense-cell direction-of-effect synthesis, focused risk-of-bias contextualisation, research-priority ranking, a *post hoc* audit of immune-mechanism integration and priority-ranking robustness analyses. Reporting and synthesis procedures were informed by PRISMA 2020, PRISMA-ScR, SWiM and OHAT guidance, but the study was designed as a priority-based mapping review rather than a conventional exhaustive full-text systematic review of all retained records.

Record flow was documented using a PRISMA 2020-style structure adapted for a priority-based evidence map, with PRISMA-ScR principles considered where relevant. In this design, a broad deduplicated corpus was screened, after which records were prioritised for focused coding according to predefined relevance and methodological criteria. Records retained in the broader screening pool but not prioritised for focused coding were shown explicitly as a deferred branch rather than being counted as exclusions.

Prospective protocol registration was not undertaken before screening. A retrospective OSF registration was completed to document the final research question, eligibility criteria, search strategy, prioritisation framework and analysis plan, and is available at https://doi.org/10.17605/OSF.IO/UVX93. A versioned protocol description, search strategies, eligibility rules, screening and coding decisions, harmonisation dictionary, audit logs and analysis materials are available in the Zenodo repository cited in the Data availability statement.

### 2.2 Data sources and search strategy

The Web of Science Core Collection and Scopus were searched and exported on 18 May 2026. Database filters restricted records to English-language article/review records published from 2005 to 2025. Review records were exported to maximise retrieval sensitivity, support audit and reference tracking, and preserve the database search trail; non-original publications were excluded during eligibility assessment. The exact database-specific search strings, filters, export fields and record counts are reported in the **Supplementary Methods** and in the Zenodo repository cited in the Data availability statement.

The search strategy combined terms for AIRDs with terms for environmental exposures. AIRD terms covered rheumatoid arthritis, systemic lupus erythematosus, lupus nephritis, Sjögren’s syndrome, systemic sclerosis, idiopathic inflammatory myopathies, dermatomyositis, polymyositis, ANCA-associated vasculitis, antiphospholipid syndrome, axial spondyloarthritis, ankylosing spondylitis, psoriatic arthritis, mixed connective tissue disease, undifferentiated connective tissue disease and adult-onset Still disease. Environmental exposure terms covered air pollution, particulate matter, traffic-related and indoor air pollution, tobacco and smoking, silica and occupational dusts, pesticides, solvents, volatile organic compounds, benzene, formaldehyde, trichloroethylene, polycyclic aromatic hydrocarbons, heavy metals, trace elements, phthalates, bisphenols, PFAS, persistent organic pollutants, polychlorinated biphenyls, dioxins, endocrine disruptors, occupational chemical exposures, mixed exposures and exposome-related terms.

The final Scopus export contained 11,916 records after applying article/review and English-language filters, and the final Web of Science Core Collection export contained 5,173 English-language article/review records. Full database-specific search strings, field tags, Boolean operators, date limits, export settings and sensitivity-search details are provided in the **Supplementary Methods** and in the Zenodo repository cited in the Data availability statement. The final analytic publication window was 2005-2025; temporal analyses were interpreted only within this complete window, and no inference was made for publication years beyond 2025.

### 2.3 Corpus import, field harmonisation and deduplication

Records exported from the two databases were imported into a structured data-processing pipeline, and database-specific fields were harmonised for bibliographic and screening variables, including title, abstract, authors, source title, publication year, DOI, database identifier, document type, language and keyword fields.

The combined pre-deduplication corpus contained 17,089 records, comprising 5,173 from the Web of Science Core Collection and 11,916 from Scopus. Automated deduplication removed 4,592 duplicate records, leaving a frozen deduplicated corpus of 12,497 records for title and abstract screening. Deduplication used a hierarchical strategy based on DOI exact matches, normalised-title matching, and bibliographic checks using author, year and source fields; candidate duplicate pairs that did not meet automatic deletion criteria were retained for audit rather than removed manually during downstream screening. Detailed deduplication rules are provided in the **Supplementary Methods**.

### 2.4 Eligibility criteria

Eligible studies were original human studies that evaluated at least one environmental exposure in relation to an AIRD population, outcome, phenotype, clinical feature, serological feature or disease-related measure.

Eligible environmental exposures included chemical, particulate, occupational and tobacco-related exposures, namely air pollution, particulate matter, traffic-related air pollution, indoor air pollution, tobacco smoke, silica, mineral dust, occupational dust, pesticides, solvents, volatile organic compounds, persistent organic pollutants, PFAS, heavy metals, trace elements, endocrine-disrupting chemicals and related environmental contaminants.

Eligible AIRD targets included rheumatoid arthritis, systemic lupus erythematosus, lupus nephritis, primary Sjögren’s syndrome, systemic sclerosis, idiopathic inflammatory myopathies, vasculitis, antiphospholipid syndrome, mixed or undifferentiated connective tissue disease, systemic autoimmune rheumatic diseases and spondyloarthritis-spectrum diseases when analysed within the autoimmune or inflammatory rheumatic disease scope.

Records were excluded if they were non-original publications, non-human or basic mechanistic studies without a human exposure–disease link, case reports or small case series, studies without an eligible environmental exposure, studies without an eligible AIRD population or outcome, studies in which AIRD was only a covariate or risk factor for an unrelated outcome, or drug/treatment-exposure studies. Broad lifestyle, social and general dietary factors without a clearly defined and extractable environmental/exposome exposure–AIRD relationship were excluded. However, specific diet-related exposures were retained when they fell within the predefined diet-related environmental/exposome family and provided an extractable exposure–AIRD association. This allowed chemically or biologically defined diet-related exposures to be mapped without broadening eligibility to general lifestyle or social determinants.

Smoking and tobacco were included only when analysed as primary environmental exposures in relation to AIRD occurrence, prevalence, activity, clinical phenotype, serology or related outcomes. Smoking variables used only as covariates, risk-score components, baseline descriptors or confounders were not considered eligible exposure–outcome links for the focused map.

### 2.5 Title and abstract screening

All 12,497 deduplicated records underwent title and abstract screening. Before formal screening, reviewers performed calibration on a pilot set to align eligibility rules and coding terminology. Title/abstract screening was performed independently by two reviewers, with records classified as include, exclude or uncertain. Records were excluded only when the title/abstract clearly indicated non-relevance; records with clear or possible relevance were retained for further prioritisation. Disagreements were resolved by consensus, with senior adjudication when needed.

Title/abstract screening excluded 4,772 records, and the remaining 7,725 records formed the broader screening pool. This pool comprised high-confidence title/abstract includes together with uncertain records retained under a conservative screening strategy.

### 2.6 Priority-based focused full-text coding strategy

Because this project was designed as a priority-based evidence map rather than an exhaustive full-text systematic review of all potentially adjacent records, the 7,725 retained records were stratified using a predefined prioritisation strategy designed to preserve the broader search trail while concentrating focused coding on records most likely to contribute direct environmental exposure-AIRD evidence.

Operationally, a direct link required the title/abstract to identify an eligible environmental exposure and an eligible AIRD population, outcome or phenotype within the same human study, with a design likely to permit extraction of the exposure–AIRD contrast. Records were also prioritised when a boundary-sensitive direct link could not be excluded. Indirect or adjacent records included mechanistic studies without an eligible human AIRD population or outcome, AIRD studies in which the environmental exposure was only a covariate or background descriptor, and exposure-focused studies without an extractable AIRD measure. Such records could inform biological interpretation through cited literature but did not contribute to evidence-map counts. Deferred records were retained in the search trail and PRISMA-style flow diagram and were not treated as formal exclusions.

Prioritisation was based on title/abstract relevance to the exposure-AIRD mapping question and did not use reported effect direction, statistical significance, journal impact, citation count or expected priority score.

The final prioritisation strata were a focused priority set (2,632 records), a secondary deferred set (3,669 records) and an additional deferred set retained for later specialised review (1,424 records). Thus, 5,093 records were retained in the broader screening pool but deferred from focused coding. These deferred records were not counted as exclusions; they were shown explicitly in the PRISMA-style flow diagram as records retained in the broader pool but not coded for the priority-based map. This design avoided conflating deferred lower-priority records with formal exclusions.

### 2.7 Full-text retrieval and eligibility assessment

Full texts were sought for all 2,632 records in the focused full-text priority set. Three reports could not be retrieved after institutional and bibliographic searches and were separated as reports not retrieved in the PRISMA flow.

The remaining 2,629 reports were assessed for eligibility. After full-text assessment, 2,196 reports were excluded; the largest exclusion categories were no eligible exposure-AIRD outcome link and not an eligible environmental exposure. A total of 433 studies were included in the final priority-coded evidence map.

Eligibility assessment and exclusion-reason coding followed a reviewer-verification workflow. A primary reviewer recorded eligibility decisions and exclusion reasons using the structured template, and a second reviewer independently checked all included studies, all boundary or conservative-exclusion records, all full-text-unavailable records, and records with uncertain or potentially overlapping exposure/outcome classification. Disagreements and boundary cases were adjudicated by consensus before the final analytic dataset was frozen. For retained records with limited full-text auditability, the audit trail preserved a specific flag and those records were excluded in the restricted-auditability sensitivity denominator.

For audit purposes, the internal full-text decision table retained the three non-retrieved reports as conservative exclusions; for PRISMA reporting, these three records were separated before the eligibility-assessment step. The final PRISMA counts were therefore 2,632 reports sought for retrieval, 3 not retrieved, 2,629 assessed for eligibility, 2,196 excluded after full-text assessment and 433 studies included.

### 2.8 Quality-control remediation and final dataset freeze

During quality control, part of the focused priority set was identified as requiring additional verification because full-text confirmation had not been uniformly documented. To prevent misclassification and preserve auditability, a documented remediation was performed before freezing the final analytic dataset. This remediation included strict rechecking of smoking and tobacco-related records, full-text verification of open-access or readily retrievable records, and targeted verification checks for records with limited full-text access. Records clearly failing the final eligibility rules were moved from include to exclude; records for which full text remained unavailable were treated as conservative exclusions where eligibility could not be supported; and boundary-sensitive retained records were explicitly flagged rather than silently merged into the core set. Where full-text auditability remained limited for selected descriptive fields, that limitation was retained in the audit trail and addressed through the restricted-auditability sensitivity denominator.

The resulting final quality-controlled analytic dataset contained 2,632 focused-priority records, of which 433 were retained in the priority-coded evidence map and 2,199 were not included, comprising 2,196 full-text exclusions and 3 reports not retrieved. None remained uncertain. This dataset was used for all downstream descriptive analyses, figures and manuscript tables.

### 2.9 Data extraction and coding

For each retained study, we extracted bibliographic, epidemiological and methodological information into a structured coding template. Variables included record identifier, title, publication year, journal, DOI, country or region, study design, population type, sample size, AIRD target, exposure domain, specific exposure agent, exposure matrix or biomarker, outcome domain, outcome measure and modelling or adjustment features. Immune-related fields included autoantibody or serologic phenotype, molecular or biomarker outcomes, genetic or epigenetic susceptibility and exposure-linked immune measurements when reported. Where a descriptive field could not be fully verified from accessible full text, its source status was recorded and the study was flagged for the restricted-auditability sensitivity denominator.

Methodological features were coded to indicate whether each study reported interaction analysis, sensitivity analysis, nonlinear analysis, mixture-method analysis, weighting or complex-survey design, and other high-value epidemiological signals. These variables were intended to characterise the design and analytical structure of the literature; they were not used as a formal risk-of-bias tool and should not be interpreted as field-wide prevalence estimates beyond the priority-coded subset.

Several variables were non-mutually exclusive: a single study could contribute to more than one exposure domain, disease category, country or methodological signal. Consequently, counts for these variables may exceed 433 and percentages may sum to more than 100%.

### 2.10 Harmonisation of exposure, AIRD and display terms

Raw exposure, AIRD and methodological labels were harmonised using an auditable display-term dictionary to improve the interpretability of figures and tables while preserving record-level coding, source-status flags and eligibility decisions.

Display-only harmonisation was used when labels required standardisation for figure rendering but did not require any change to eligibility, grouping rules or denominator. For example, PFAS-related labels were displayed under PFAS/persistent organic pollutants, and solvent- or VOC-related labels under VOCs/organic solvents, after record-level checks verified that these merges did not create overlapping counts. AIRD display-label refinements, including abbreviation formatting such as axSpA, were also applied only at the display layer. No display-label patch changed inclusion decisions, full-text decisions, denominator, raw coding or record-level grouping.

### 2.11 Evidence mapping and descriptive synthesis

The main evidence map summarised the distribution of included studies across environmental exposure families and AIRD groups. Exposure families included smoking and tobacco; air pollution and ambient exposures; occupational exposures; metals; pesticides; solvents, VOCs and persistent organic pollutants; endocrine disruptors and additives; and diet-related chemicals and other eligible environmental agents retained after eligibility review. AIRD groups included rheumatoid arthritis, systemic lupus erythematosus, systemic sclerosis, primary Sjögren’s syndrome, spondyloarthritis-related diseases, idiopathic inflammatory myopathy, vasculitis and other or unspecified autoimmune rheumatic disease categories.

We generated descriptive distributions for exposure domains, AIRD targets, study designs, methodological signals, countries or regions, publication years and source journals, and cross-tabulated exposure families by AIRD categories to identify evidence-rich, limited-evidence and no-mapped-study combinations within the priority-coded set. Cell counts in these matrices represent distinct studies contributing to each exposure-disease combination; because studies could address multiple exposures and diseases, cells were not mutually exclusive and could sum to more than 433.

We did not pool effect estimates because exposure definitions, outcome definitions, study populations, designs and modelling approaches were heterogeneous. The synthesis was therefore descriptive and structural rather than meta-analytic.

### 2.12 Direction-of-effect synthesis and focused risk-of-bias assessment

After the main evidence map had been frozen, we selected four high-density exposure–AIRD cells for an additional descriptive synthesis: smoking/tobacco exposure × rheumatoid arthritis, air pollution/ambient exposure × rheumatoid arthritis, air pollution/ambient exposure × systemic lupus erythematosus, and occupational exposure × systemic sclerosis. These cells were chosen, from among the most populated combinations in the focused map, to span the principal exposure families (tobacco, ambient air pollution and occupational exposures) and the most studied connective-tissue and inflammatory-arthritis diseases, and to include the long-investigated occupational–systemic sclerosis relationship. They were not intended as the four single largest cells by count; rather, they were selected to provide disease- and exposure-diverse, sufficiently populated cells suitable for structured direction-of-effect evaluation.

Direction-of-effect coding followed a SWiM-aligned framework and was performed at the study-cell level rather than the article level (27). For each study-cell entry, reviewers identified the dominant exposure–outcome contrast using prespecified rules: primary outcomes were prioritised when stated; otherwise, AIRD occurrence, incidence or prevalence was prioritised, followed by disease activity, severity, phenotype, serological status or other clinically relevant AIRD outcomes. When multiple models were reported, the fully adjusted model was preferentially used. Final direction categories were positive/harmful, inverse/protective, null/no clear association, mixed, and not classifiable. Not-classifiable entries were retained in the audit trail but were not interpreted as directional votes.

A focused OHAT-style risk-of-bias assessment adapted for environmental epidemiology was conducted for the same study-cell entries (28, 29). The domains considered were exposure assessment, AIRD outcome ascertainment, confounding control, selection or population definition, missing data or attrition, and selective reporting or analytical transparency. Overall judgements were lower risk, some concerns, higher risk, unclear, or not assessed. The not-assessed category was reserved for entries retained in the audit trail but not suitable for a meaningful cell-specific judgement, such as entries without sufficient target-cell results or entries not classifiable for direction. Two reviewers independently performed full-text verification, direction coding and focused risk-of-bias assessment, and disagreements were resolved by consensus adjudication. Inter-reviewer agreement was quantified using Cohen’s κ for direction coding, for each focused risk-of-bias domain and for the overall focused risk-of-bias judgement (30). This layer was used to contextualise directional consistency and was not intended to generate pooled effect estimates, a full OHAT certainty assessment or formal certainty-of-evidence grades.

### 2.13 Research-priority scoring framework

To translate the evidence map into future research priorities, we constructed a consensus scoring framework across the 64 exposure-family × AIRD cells defined by eight exposure families and eight AIRD groups. The framework was designed to distinguish simple absence or scarcity of evidence from higher-priority research opportunities where sparse evidence overlapped with plausible mechanisms, public-health relevance and disease burden.

**Figure 1:**
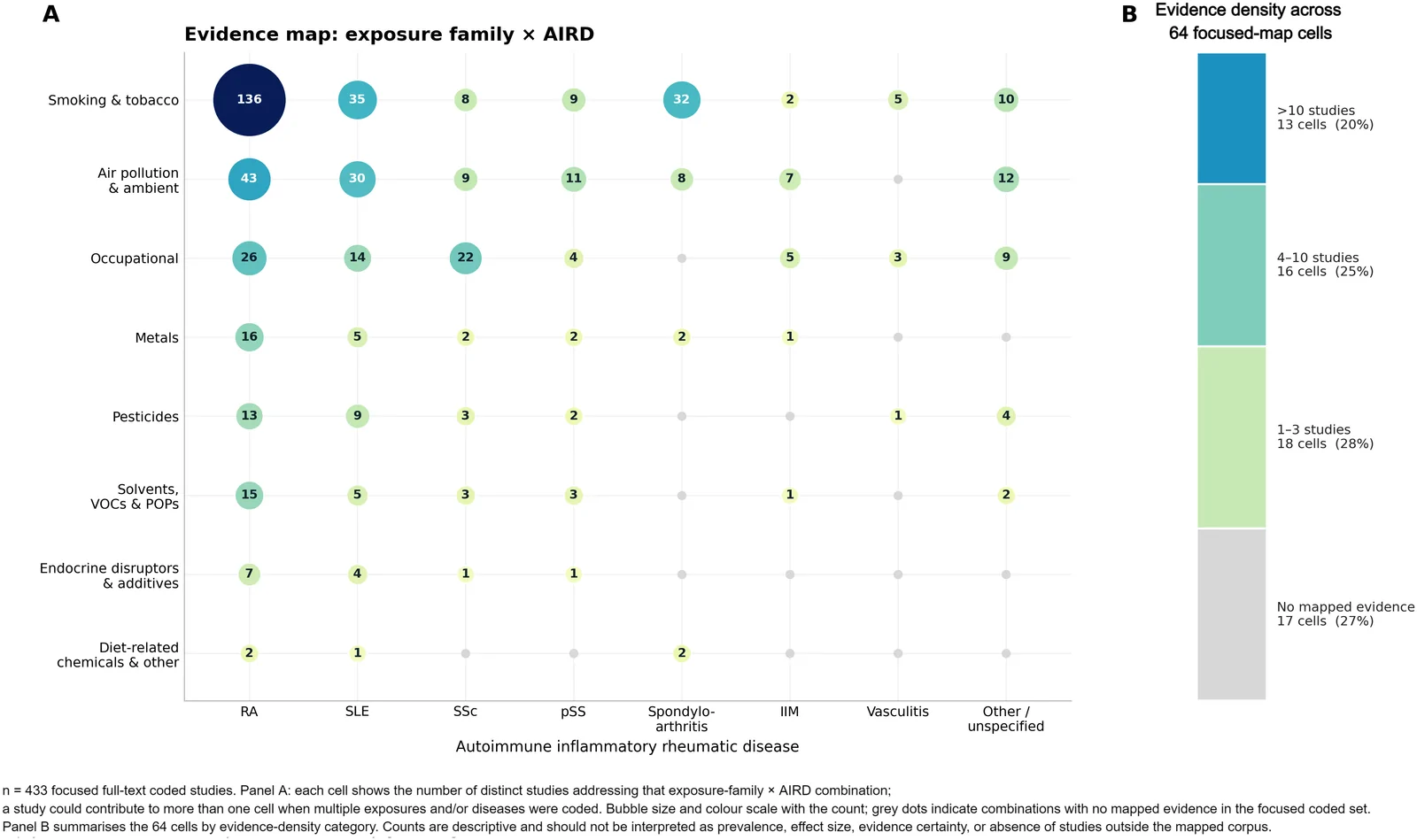
Evidence map of environmental exposure family by AIRD. **(A)** Bubble matrix of the number of distinct studies addressing each exposure-family × AIRD combination. Bubble size and colour scale with study count; grey dots mark combinations with no mapped study in the priority-coded set. A study may cover several exposures and/or diseases and can therefore appear in more than one cell, so cell counts sum to more than 433. **(B)** summarises the 64 cells by evidence-density category. Spondyloarthritis groups ankylosing spondylitis, axial spondyloarthritis, spondyloarthritis and psoriatic arthritis; IIM includes antisynthetase syndrome. “No mapped study” refers only to the priority-coded set and does not imply absence of association or absence of any study outside this map.

Three reviewers independently scored each cell across four 0–5 dimensions: scarcity-adjusted evidence gap, exposure prevalence or public-health relevance, biological plausibility, and AIRD burden or unmet need. Biological plausibility was operationalised through prespecified pathway domains, including mucosal or epithelial barrier hypotheses, oxidative stress, post-translational protein modification, epigenetic or endocrine immune modulation, innate/adaptive immune dysregulation, autoantibody formation and disease-specific immunopathology. The scarcity dimension incorporated expected evidence volume within the 64-cell matrix, allowing a cell-level gap to be distinguished from globally sparse disease rows or exposure columns. The consensus score was the sum of median reviewer scores; cells with a reviewer spread of at least 2 points in any dimension were adjudicated. Agreement was quantified using two-way random-effects absolute-agreement ICC(2,k) (31) and the proportion of scores within ±1 point. Detailed scoring anchors are provided in **Supplementary Table 12**. *Post hoc* robustness analyses recalculated rankings after omitting each dimension in turn, halving the weight assigned to biological plausibility and doubling the weight assigned to scarcity. Spearman rank correlation and top-15 overlap were compared with the original four-dimension ranking. During revision, the current-evidence counts used for the 64-cell priority display were reconciled with the finalised 8×8 evidence matrix after contextual non-AIRD labels were removed from the Other/unspecified column in an intermediate reconstruction. This reconciliation affected current-evidence counts in the affected Other/unspecified cells but reproduced the submitted **Figure 1** matrix and did not alter the principal non-Other priority cells. Scores indicate research-planning priority, not association strength, certainty or causality.

### 2.14 *Post hoc* immune-mechanism integration audit

In response to peer review, we conducted a *post hoc* structured audit of immune-mechanism integration within the 433-study map. Each study was assigned to the highest applicable level of a four-level hierarchy: Level 3, direct measurement of a pathway-proximal mechanistic intermediary or omics readout; Level 2, direct measurement of an immune, serologic, complement or inflammatory biomarker; Level 1, analysis of an immune-defined disease subtype or genetic susceptibility/interaction without a measured intermediary; or Level 0, no integrated exposure-linked immune endpoint. Classification was based on the reported exposure–outcome analysis rather than on mechanistic background statements; routine use of an autoantibody solely for case definition was not sufficient for Level 2.

The audit used the frozen structured fields, titles, abstracts and available full-text or result-table verification notes. Records with pathway-proximal measurements, boundary status or limited auditability were prioritised for targeted author verification. All 69 priority sign-off records were subsequently reviewed and approved by the authors before the revised counts were frozen. We summarised the hierarchy overall, by exposure family, by AIRD group and in the four dense cells (**Table 1**; **Supplementary Tables 14, 15A, 15B**). A sensitivity analysis excluded the 11 authoritative boundary records. The hierarchy measures the degree of immune integration and does not grade biomarker validity, mechanism certainty or causality.

**Table 1:** *Post hoc* audit of immune-mechanism integration in the overall map and four dense exposure–AIRD cells.

Population/cell	Total n	Level 3	Level 2	Level 1	Level 0
Overall map	433	25	79	63	266
Smoking/tobacco × RA	136	6	40	39	51
Air pollution × RA	43	1	3	3	36
Air pollution × SLE	30	3	3	0	24
Occupational × SSc	22	1	5	1	15

Level 3 = direct pathway-proximal mechanistic intermediary or omics readout; Level 2 = direct immune/serologic, complement or inflammatory biomarker; Level 1 = immune-defined subtype or genetic susceptibility/interaction without a measured intermediary; Level 0 = no integrated exposure-linked immune endpoint. Levels are mutually exclusive and assigned at the study level. RA, rheumatoid arthritis; SLE, systemic lupus erythematosus; SSc, systemic sclerosis.

### 2.15 Secondary publication descriptors

Secondary descriptive analyses characterised publication year, country/region and source-journal distributions within the priority-coded evidence base. These outputs were included to provide contextual information only and did not determine evidence density, directionality, focused risk-of-bias judgements or research-priority scores. A supplementary author-keyword co-occurrence display was generated among studies with available author keywords after light normalisation for spelling and display consistency. It was interpreted only as a descriptive aid to recurring topics, not as a formal citation-network analysis.

Cited-reference, co-citation and bibliographic-coupling analyses were not conducted. This study is presented as a record-level priority-based evidence map with focused synthesis, rather than as a bibliometric analysis.

### 2.16 Sensitivity scope assessment

We prespecified nested sensitivity denominators to make the scope and auditability constraints of the priority-coded map transparent. The main map used all 433 retained studies. Sensitivity denominator 1 excluded 11 authoritative boundary records, yielding n = 422. Sensitivity denominator 2 excluded 141 retained records with limited full-text auditability and metadata-assisted descriptive coding, yielding n = 292. The boundary and restricted-auditability lists had zero overlap, so the combined denominator was n = 281.

These nested denominators define alternative, more conservative analytic frames; they do not create new eligibility decisions, meta-analytic datasets or causal estimates. Main figures and primary descriptive results are based on the frozen main-analysis set of 433 studies. The sensitivity framework is intended to delimit the scope of inference and facilitate auditable follow-up analyses, rather than to imply that the focused map is an exhaustive field-wide count.

### 2.17 Software and reproducibility

Data processing, screening-table construction, tabulation, harmonisation and primary figure preparation were conducted in R version 4.5.1 using RStudio version 2025.5.0.496 on Windows 11. Key R packages included tidyverse 2.0.0, dplyr 1.2.0, tidyr 1.3.2, readr 2.2.0, readxl 1.4.5, openxlsx 4.2.8.1, writexl 1.5.4, stringr 1.6.0, lubridate 1.9.5, janitor 2.2.1, igraph 2.2.2, ggplot2 4.0.2, ggrepel 0.9.8, scales 1.4.0, patchwork 1.3.2, RColorBrewer 1.1-3, forcats 1.0.1, purrr 1.2.1, tibble 3.3.1, data.table 1.18.2.1., haven 2.5.5, flextable 0.9.11. The *post hoc* mechanism-integration audit and priority-ranking robustness checks were additionally implemented in Python 3.13.5 using pandas 2.2.3 and openpyxl 3.1.5. Session information, analysis workbooks and software versions were exported for audit and are available in the repository cited in the Data availability statement.

All major processing stages were versioned and checked using step-specific logs. The final analysis used the quality-controlled analytic dataset together with the final harmonised analysis tables and figure datasets; earlier superseded preliminary datasets were not used.

## 3 Results

### 3.1 Study selection and construction of the focused evidence map

The database searches identified 17,089 records before deduplication, including 5,173 from the Web of Science Core Collection and 11,916 from Scopus. After automated deduplication, 12,497 records remained for title and abstract screening, of which 4,772 were excluded at the title/abstract stage, leaving 7,725 records in the broader screening pool.

**Figure 2:**
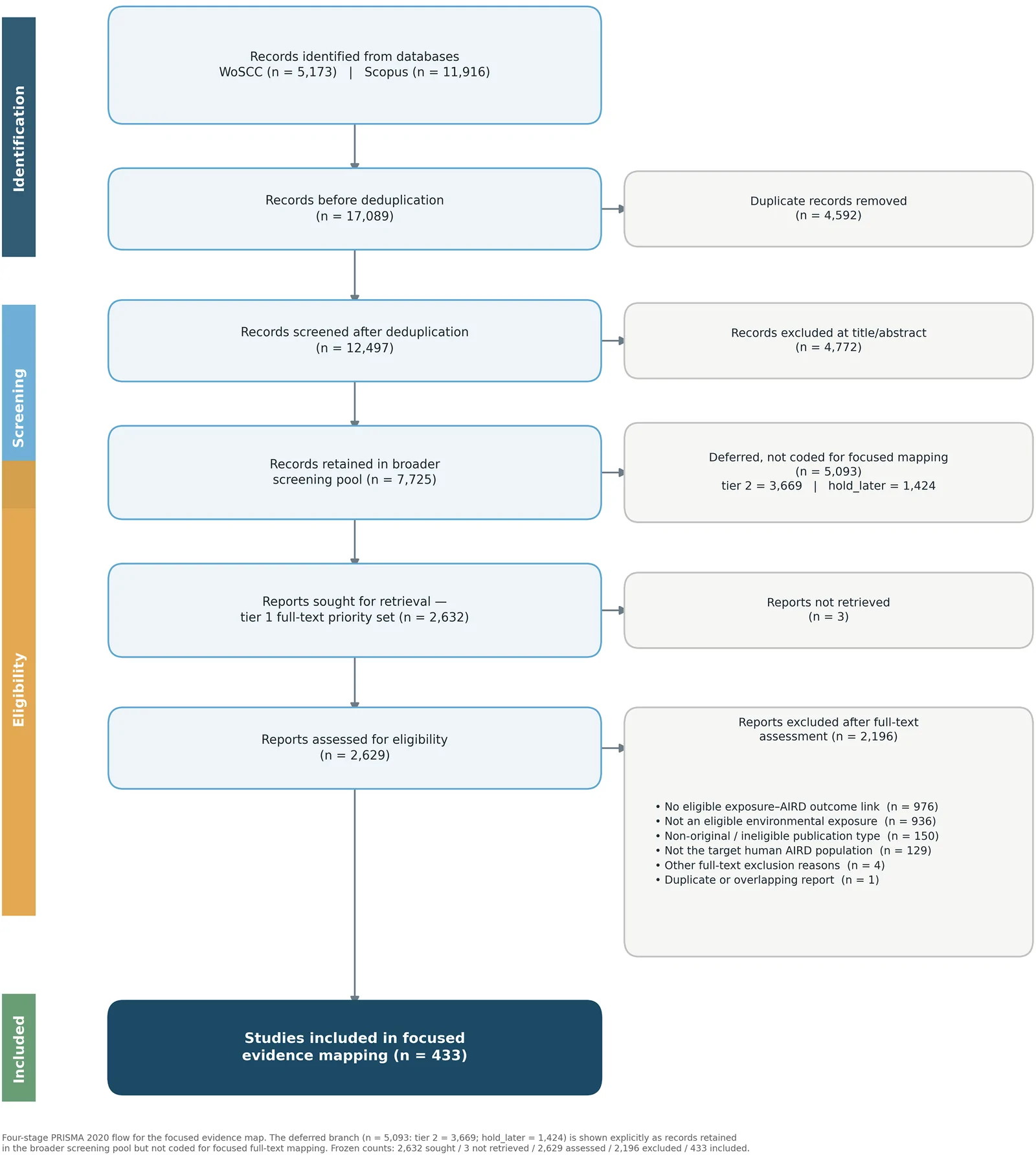
PRISMA 2020-style flow diagram. Four-stage flow of records through identification, screening, eligibility and inclusion for the priority-based evidence map. Of 17,089 records before deduplication (Web of Science Core Collection n = 5,173; Scopus n = 11,916), 4,592 duplicates were removed, 4,772 were excluded at title/abstract, and 7,725 entered the broader screening pool. A deferred branch of 5,093 records (secondary deferred set = 3,669; additional deferred set retained for later specialised review = 1,424) was retained in the pool but not coded for the priority-based map. Of 2,632 focused-priority reports sought for retrieval, 3 were not retrieved, 2,629 were assessed, and 2,196 were excluded after full-text assessment, leaving 433 studies included.

The retained records were then stratified for focused evidence mapping. A total of 2,632 records were selected for full-text retrieval and focused coding, whereas 5,093 records were retained in the broader screening pool but deferred from focused coding. Among the 2,632 reports sought for retrieval, three could not be retrieved; the remaining 2,629 reports were assessed for eligibility, of which 2,196 were excluded after full-text assessment. The final priority-coded evidence map included 433 studies (**Figure 2**).

The leading full-text exclusion categories were no eligible exposure–AIRD outcome link (n = 976), not an eligible environmental exposure (n = 936), non-original study or ineligible publication type (n = 150), not the target human AIRD population (n = 129), other full-text exclusion reasons (n = 4), and duplicate or overlapping report (n = 1). Characteristics of included studies are summarised in **Table 2**.

**Table 2:** Characteristics of included studies (n = 433).

Characteristic	Category	Studies, n	%
AIRD (disease) — not mutually exclusive			
	RA	254	58.7
	SLE	101	23.3
	SSc	48	11.1
	pSS	32	7.4
	Spondyloarthritis (AS/axSpA/SpA/PsA)	42	9.7
	IIM (incl. antisynthetase)	14	3.2
	Vasculitis (incl. AAV)	9	2.1
	Other/unspecified	37	8.5
Environmental exposure family — not mutually exclusive			
	Smoking & tobacco	215	49.7
	Air pollution & ambient	83	19.2
	Occupational	53	12.2
	Metals	27	6.2
	Pesticides	23	5.3
	Solvents, VOCs & POPs	25	5.8
	Endocrine disruptors & additives	12	2.8
	Diet-related chemicals & other eligible exposures	5	1.2
Exposure coding breadth			
	One coded exposure domain/agent	69	15.9
	Two or more coded exposure domains/agents	364	84.1
Study design			
	Cohort	162	37.4
	Observational study, not further specified	84	19.4
	Case-control	81	18.7
	Cross-sectional	60	13.9
	Comparative risk assessment/burden modelling	29	6.7
	Time-series	8	1.8
	Mendelian randomisation	5	1.2
	Case-crossover	2	0.5
	Exposure assessment/tool development	2	0.5
Methodological signal (yes) — not mutually exclusive			
	Interaction analysis	160	37.0
	Sensitivity analysis	50	11.5
	Weighting or complex survey design	39	9.0
	Nonlinear analysis	38	8.8
	Mixture method	21	4.8
Country/region — multi-country studies counted in each			
	United States	131	30.3
	Not reported	91	21.0
	Others	79	18.2
	Sweden	34	7.9
	United Kingdom	34	7.9
	China	29	6.7
	Canada	17	3.9
	Europe	17	3.9
	Global	14	3.2
	Italy	12	2.8
	France	11	2.5
Publication year			
	2005–2009	45	10.4
	2010–2014	66	15.2
	2015–2019	116	26.8
	2020–2024	158	36.5
	2025	48	11.1
Sample size			
	Reported as a single extractable n	244	56.4
	Median (IQR), among studies reporting	426 (119–1,475)	

Counts are numbers of studies in the n = 433 priority-coded map; percentages use the denominator n = 433. Disease and exposure categories are not mutually exclusive, so sub-totals can exceed 433 and percentages can exceed 100%. Exposure coding breadth refers to the number of exposure domains or agents coded for mapping purposes and should not be interpreted as formal mixture modelling; formal mixture-method analysis was separately coded as a methodological signal. Abbreviations: RA, rheumatoid arthritis; SLE, systemic lupus erythematosus; SSc, systemic sclerosis; pSS, primary Sjögren’s syndrome; IIM, idiopathic inflammatory myopathy; AAV, ANCA-associated vasculitis; AIRD, autoimmune inflammatory rheumatic disease; IQR, interquartile range. “Others” aggregates remaining individual countries each contributing few studies.

### 3.2 Distribution of environmental exposure domains

**Figure 3:**
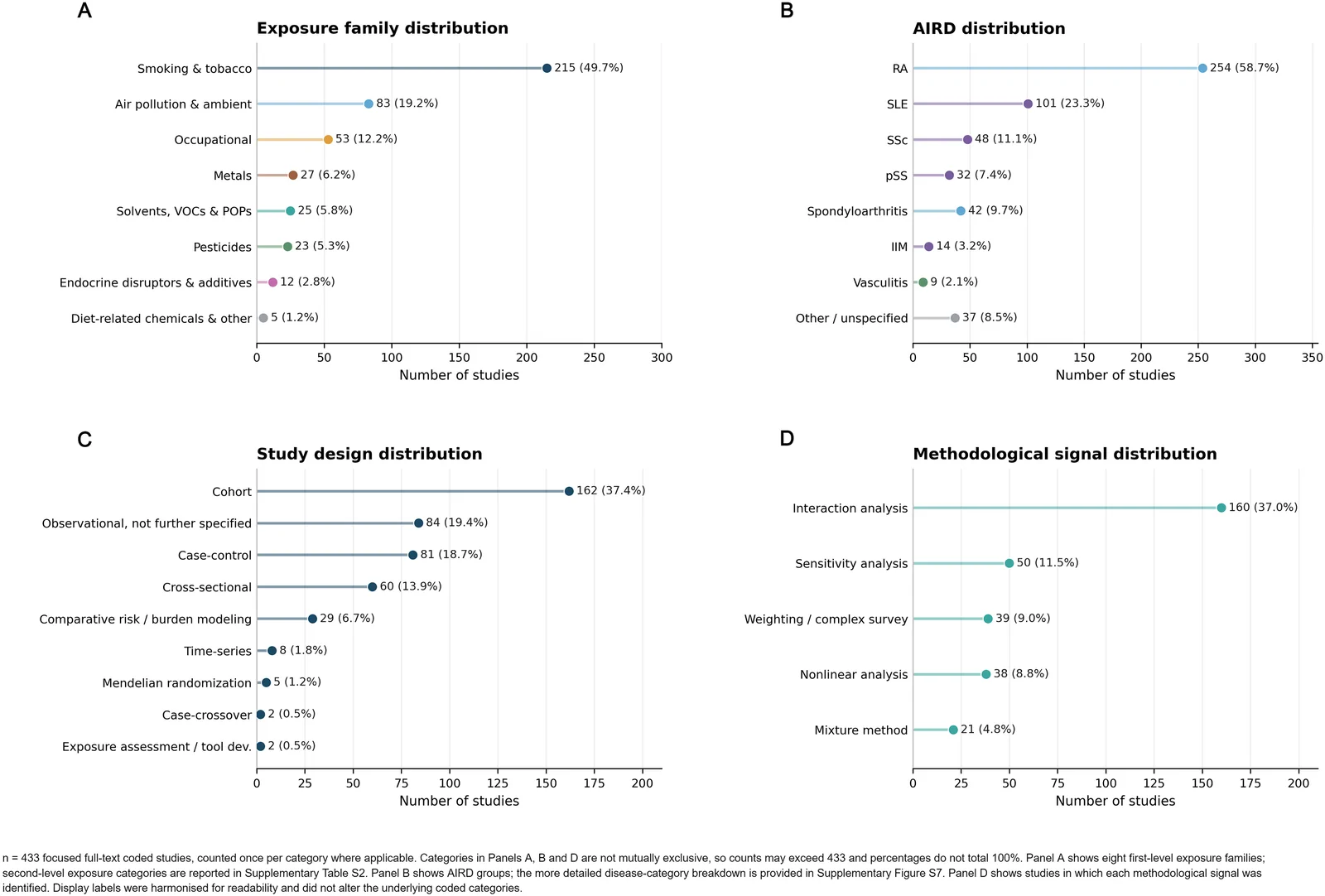
Composition of the priority-coded evidence map. **(A)** Number of included studies per first-level exposure family, each study counted once per family. **(B)** Number of included studies per harmonised AIRD group. **(C)** Distribution of study designs. **(D)** Distribution of methodological signals reported within the priority-coded subset. Categories in panels **(A, B, D)** are not mutually exclusive, so counts can sum to more than 433 and percentages do not total 100%; in panel C each study is assigned a single primary design. The detailed second-level exposure categories are reported in **Supplementary Table 2** and the detailed 18-category AIRD breakdown in **Supplementary Figure 1**. AIRD, autoimmune inflammatory rheumatic disease; RA, rheumatoid arthritis; SLE, systemic lupus erythematosus; SSc, systemic sclerosis; pSS, primary Sjögren’s syndrome; IIM, idiopathic inflammatory myopathy; VOCs, volatile organic compounds; POPs, persistent organic pollutants.

Counted at the level of the eight predefined first-level exposure families, the included studies were dominated by smoking and tobacco exposure, addressed in 215 studies (49.7% of the focused evidence map; **Figure 3A**). Air pollution and ambient exposures formed the second-largest family (n = 83, 19.2%), followed by occupational exposures (n = 53, 12.2%).

The remaining families were less frequently represented: metals (n = 27, 6.2%); solvents, VOCs and persistent organic pollutants (n = 25, 5.8%); pesticides (n = 23, 5.3%); endocrine disruptors and additives (n = 12, 2.8%); and diet-related chemicals and other eligible environmental agents (n = 5, 1.2%). Within these families, specific second-level exposure categories — including particulate matter and individual gaseous pollutants, silica and occupational dusts, heavy metals and trace elements, PFAS and other persistent organic pollutants, phthalates, bisphenol A and polycyclic aromatic hydrocarbons — were each represented by only small numbers of studies and are reported in full in **Supplementary Table 2**.

Because a study could address exposures in more than one family, family counts were not mutually exclusive and summed to more than 433.

### 3.3 Distribution of AIRD categories

Rheumatoid arthritis was the most frequently studied AIRD category, appearing in 254 studies (58.7%), followed by systemic lupus erythematosus (n = 101, 23.3%), systemic sclerosis (n = 48, 11.1%) and primary Sjögren’s syndrome (n = 32, 7.4%) (**Figure 3B**). Spondyloarthritis-spectrum diseases together accounted for 42 studies (9.7%), and other or unspecified autoimmune rheumatic disease categories for 37 studies (8.5%). The detailed 18-category AIRD breakdown is provided in **Supplementary Figure 1**.

Less frequently represented categories included idiopathic inflammatory myopathy (n = 14, 3.2%), psoriatic arthritis (n = 13, 3.0%), spondyloarthritis (n = 10, 2.3%) and vasculitis (n = 9, 2.1%). Smaller categories included systemic autoimmune diseases, axial spondyloarthritis, antiphospholipid syndrome, ANCA-associated vasculitis, juvenile idiopathic arthritis or juvenile rheumatoid arthritis, palindromic rheumatism, antisynthetase syndrome and autoimmune rheumatic disease not otherwise specified.

A single study could contribute to more than one AIRD category; therefore, disease counts were non-mutually exclusive and percentages did not sum to 100%.

### 3.4 Exposure–disease evidence map

The cross-classified evidence map showed a highly uneven exposure–disease landscape (**Figure 1**). The densest evidence cell was smoking/tobacco exposure in rheumatoid arthritis (n = 136), followed by smoking/tobacco exposure in systemic lupus erythematosus (n = 35) and spondyloarthritis-related diseases (n = 32). Smoking/tobacco exposure also appeared across systemic sclerosis, primary Sjögren’s syndrome, vasculitis and other or unspecified AIRD categories, indicating that tobacco exposure was the most broadly distributed exposure family in the focused evidence base.

Air pollution and ambient exposure studies were also concentrated in rheumatoid arthritis (n = 43) and systemic lupus erythematosus (n = 30), with smaller but notable representation in primary Sjögren’s syndrome, systemic sclerosis, spondyloarthritis-related diseases, idiopathic inflammatory myopathy and other or unspecified AIRD categories. Occupational exposures were most prominent in rheumatoid arthritis (n = 26), systemic sclerosis (n = 22) and systemic lupus erythematosus (n = 14), consistent with the strong presence of silica, mineral dust and occupational inhalational exposures in these disease areas.

Evidence for pesticides, metals, solvents/VOCs/POPs and endocrine disruptors was comparatively sparse and unevenly distributed. These exposure families were most often linked to rheumatoid arthritis and systemic lupus erythematosus, with fewer studies addressing systemic sclerosis, primary Sjögren’s syndrome, idiopathic inflammatory myopathy, vasculitis or other AIRD categories. Several exposure–AIRD combinations contained only one mapped study or no mapped study within the priority-coded set, highlighting substantial evidence gaps outside the dominant smoking–rheumatoid arthritis and air-pollution–rheumatoid arthritis/SLE clusters.

Because both exposure families and AIRD categories were non-mutually exclusive, cell counts in the evidence map could sum to more than the total number of included studies.

At the level of evidence volume, smoking and occupational inhalational exposures were concentrated in disease areas for which mucosal, pulmonary, post-translational or immune–fibrotic hypotheses are biologically plausible, whereas air-pollution studies were concentrated in rheumatoid arthritis and systemic lupus erythematosus. However, evidence density did not indicate that those pathways had been directly measured. Metals, pesticides, solvents, POPs, PFAS and endocrine disruptors were less frequently mapped and were rarely studied across the full AIRD spectrum. The *post hoc* mechanism-integration audit therefore examined whether the mapped human studies tested immune or molecular endpoints rather than only reporting exposure–disease associations.

### 3.5 Immune-mechanism integration across mapped studies

Of the 433 studies, 25 (5.8%) directly measured a pathway-proximal mechanistic intermediary or omics readout (Level 3), 79 (18.2%) directly measured an immune, serologic, complement or inflammatory biomarker (Level 2), 63 (14.5%) incorporated an immune-defined disease subtype or genetic-susceptibility analysis without a measured intermediary (Level 1), and 266 (61.4%) did not integrate an immune endpoint into the exposure–AIRD analysis (Level 0; **Table 1**; **Supplementary Tables 14, 15A, 15B**). Thus, 104 studies (24.0%) directly measured an immune/serologic biomarker or mechanistic intermediary, and 167 (38.6%) contained any immune-informed component. One diet-related record (SCR_07901) was retained under the predefined exposome-oriented diet-related exposure family because it reported a specific exposure–AIRD association and explicitly analysed inflammatory markers as mediators; it was classified as Level 2 rather than Level 3.

Integration differed across exposure–disease pairs. In smoking/tobacco × rheumatoid arthritis (n = 136), the hierarchy comprised 6 Level-3, 40 Level-2, 39 Level-1 and 51 Level-0 studies. Air-pollution × rheumatoid arthritis comprised 1, 3, 3 and 36 studies, respectively; air-pollution × systemic lupus erythematosus comprised 3, 3, 0 and 24; and occupational × systemic sclerosis comprised 1, 5, 1 and 15. These distributions show that the densest smoking–rheumatoid arthritis literature contained substantially more immune-defined and biomarker studies, whereas the other dense cells predominantly reported disease occurrence, activity or clinical phenotype without direct pathway measurement (**Table 1**; **Supplementary Table 14**).

After excluding the 11 authoritative boundary records, 103 of 422 studies (24.4%) remained in Levels 2–3, 24 (5.7%) remained in Level 3 and 164 (38.9%) remained in Levels 1–3. The mechanism-integration proportions were therefore materially unchanged in the boundary-excluded frame.

### 3.6 Study design characteristics and methodological signals

The focused evidence map was dominated by observational epidemiological designs (**Figure 3C**). Cohort studies were the most common design (n = 162, 37.4%), followed by observational studies not further specified (n = 84, 19.4%), case-control studies (n = 81, 18.7%) and cross-sectional studies (n = 60, 13.9%). Comparative risk assessment or burden-modelling studies accounted for 29 studies (6.7%). Time-series designs (n = 8, 1.8%), Mendelian randomisation studies (n = 5, 1.2%), exposure-assessment or tool-development studies (n = 2, 0.5%) and case-crossover studies (n = 2, 0.5%) were uncommon.

Methodological signals were unevenly distributed across the priority-coded evidence base (**Figure 3D**). Interaction analysis was the most frequently identified signal (n = 160, 37.0%). Sensitivity analysis was reported in 50 studies (11.5%), weighting or complex survey design in 39 (9.0%), nonlinear analysis in 38 (8.8%) and mixture-method analysis in 21 (4.8%). Although 84.1% of studies addressed two or more exposure domains or agents for mapping purposes, formal mixture-modelling methods were applied in only 4.8%, indicating that multi-agent coverage rarely translated into integrated mixture analysis. These methodological signals were coded within the priority-coded subset and should not be interpreted as field-wide prevalence estimates.

### 3.7 Geographic and temporal distribution

**Figure 4:**
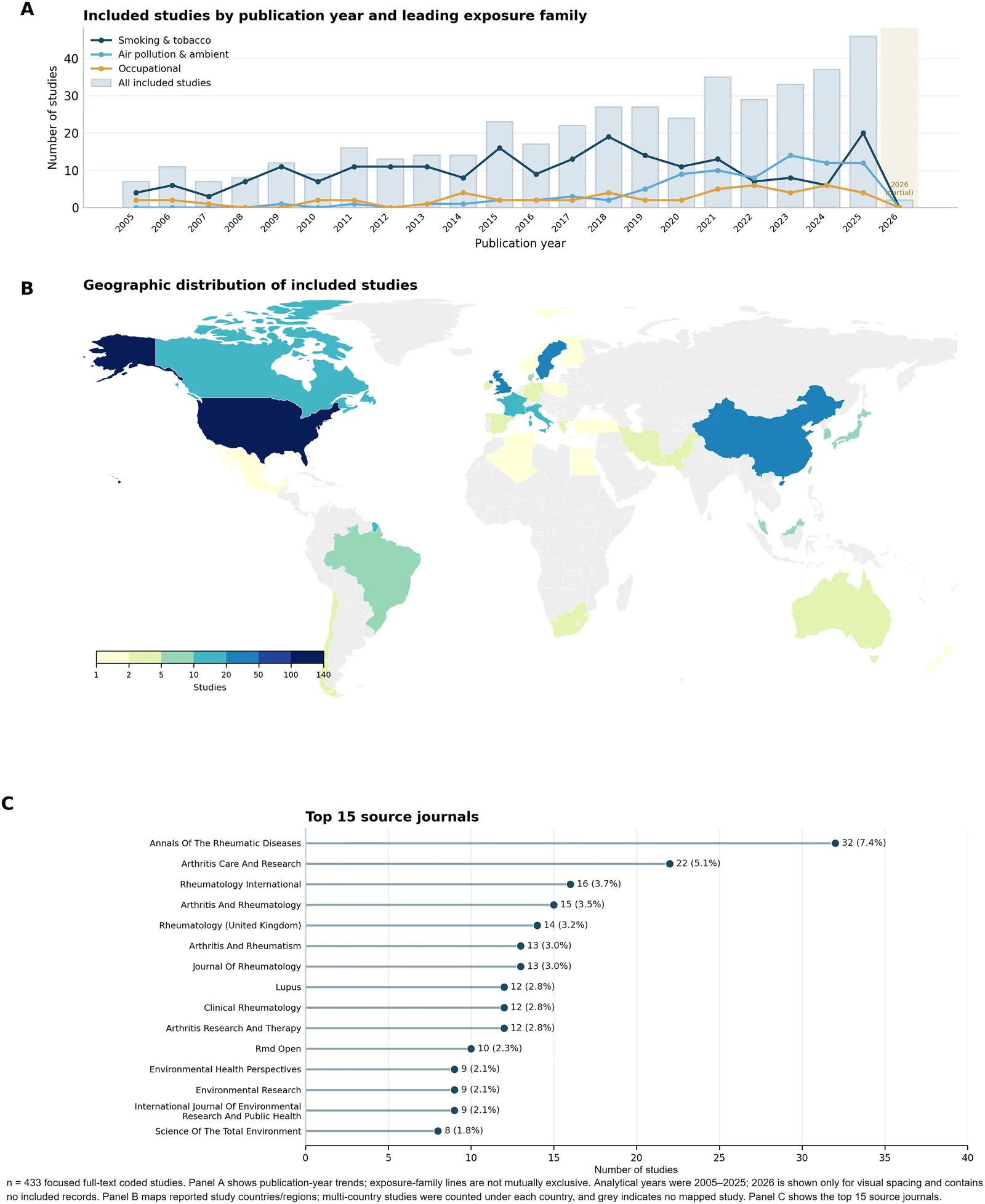
Temporal, geographic and source-journal descriptors of included studies. **(A)** Included studies by publication year and leading exposure families; bars show all included studies per year and lines show studies whose exposure set includes the indicated family (lines are not mutually exclusive and need not sum to the bar). **(B)** Choropleth map of included studies by reported country/region; multi-country studies were counted under each contributing country/region, and countries with no included study are shown in grey. **(C)** The 15 most frequent source journals among the 433 included studies across 139 journals. These descriptors are contextual and were not used in evidence-density, direction-of-effect, risk-of-bias or research-priority calculations. The final analytic publication window was 2005-2025; temporal and geographic patterns are descriptive and refer to the priority-coded map.

The geographic distribution of included studies was concentrated in a small number of countries and regions (**Figure 4B**; **Supplementary Figure 4**). The United States contributed the largest number of studies (n = 131, 30.3%), followed by Sweden (n = 34, 7.9%), the United Kingdom (n = 34, 7.9%) and China (n = 29, 6.7%). Canada and Europe-level studies each contributed 17 studies (3.9%), while global or multinational analyses contributed 14 (3.2%). Country or region information was not reported in 91 studies (21.0%). The geographic map showed 344 country attributions across 34 countries after excluding unreported and non-country labels, with the strongest concentration in North America, parts of Europe and China.

The annual number of included studies increased over the 2005–2025 publication window, with marked expansion in recent years (**Figure 4A**). Early years contributed relatively few studies, whereas annual output increased substantially after the mid-2010s and reached its highest levels in the 2020s. Smoking and tobacco-related studies were present throughout the study period, while air pollution and ambient exposure studies became more prominent in recent years. Temporal patterns should be interpreted descriptively rather than as formal trend estimates.

### 3.8 Secondary publication descriptors

Source-journal distribution and author-keyword co-occurrence were treated as contextual descriptors only. The included studies were distributed across 139 source journals, reflecting a multidisciplinary interface between rheumatology, immunology, epidemiology, environmental health and public health; these descriptors were not used to determine evidence density, direction-of-effect, risk-of-bias judgements or priority scores. Full source-journal counts and the contextual author-keyword display are provided in the **Supplementary Materials**.

### 3.9 Direction-of-effect synthesis and focused risk-of-bias assessment

**Figure 5:**
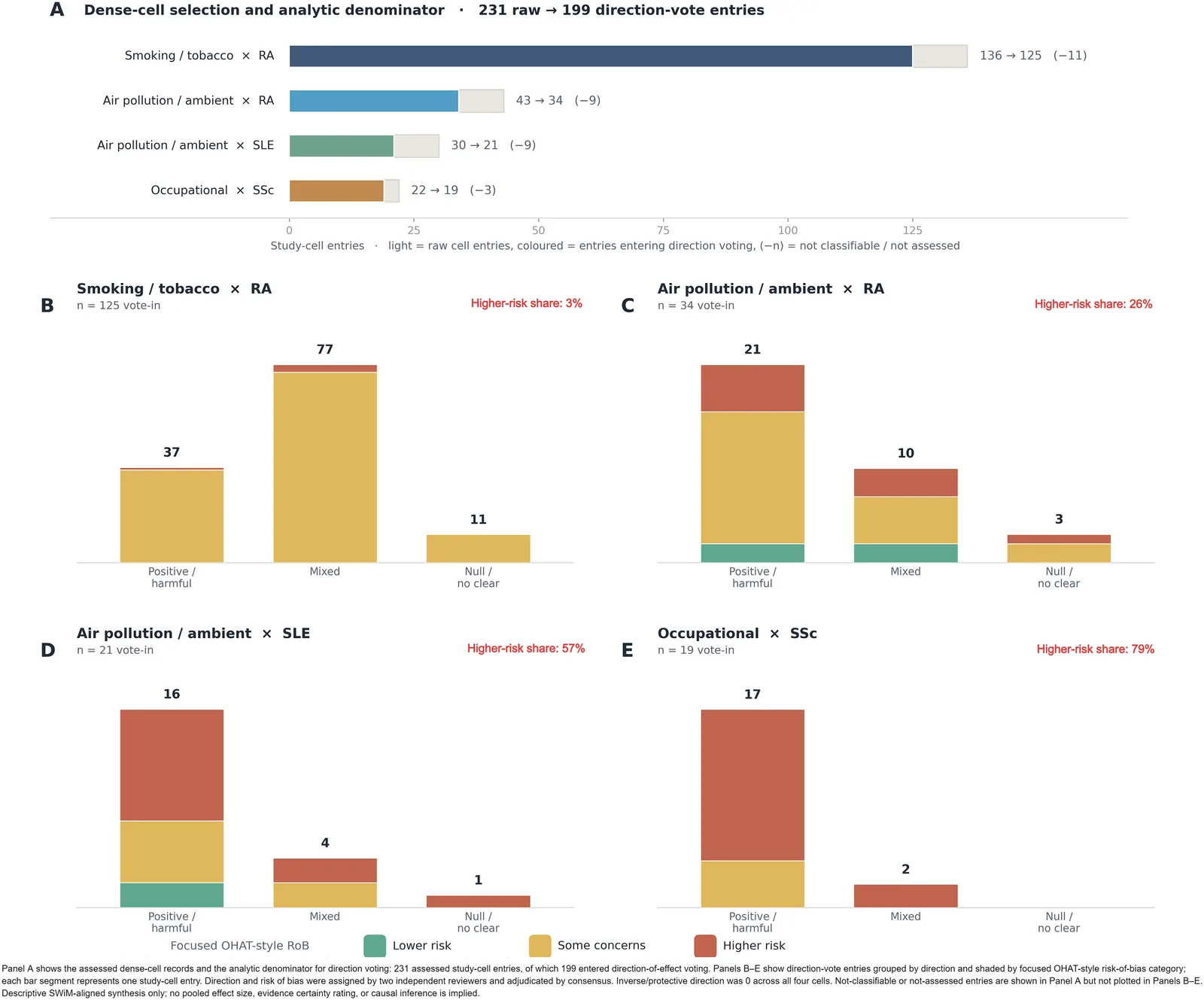
Direction-of-effect synthesis with focused risk of bias. Four selected high-density exposure-AIRD cells were re-coded at study-cell level using a SWiM-aligned direction-of-effect framework and a focused OHAT-style risk-of-bias assessment. **(A)** shows the dense-cell selection and analytic denominator (231 assessed study-cell entries; 199 entering direction voting); panels **(B–E)** show direction-vote entries grouped by direction and shaded by focused risk-of-bias category, with each tile representing one study-cell entry. Not-classifiable entries and records not assessed for risk of bias are retained in the audit trail but are not plotted as directional votes. Cell-level percentage badges indicate the share of direction-vote entries judged as higher risk of bias; they are not estimates of increased disease risk. The figure is descriptive and should not be interpreted as a pooled effect estimate, evidence-certainty grade or causal inference. Abbreviations: AIRD, autoimmune inflammatory rheumatic disease; RA, rheumatoid arthritis; SLE, systemic lupus erythematosus; SSc, systemic sclerosis; OHAT, Office of Health Assessment and Translation; RoB, risk of bias.

The four selected high-density exposure–AIRD cells comprised 231 study-cell entries: smoking/tobacco exposure × rheumatoid arthritis (n = 136), air pollution/ambient exposure × rheumatoid arthritis (n = 43), air pollution/ambient exposure × systemic lupus erythematosus (n = 30), and occupational exposure × systemic sclerosis (n = 22). Thirty-two entries were not classifiable because the target-cell result, full-text result detail or sufficient exposure–outcome information was unavailable for a robust directional judgement; these were retained in the audit trail but not interpreted as directional votes. The remaining 199 study-cell entries contributed to the direction-of-effect synthesis (**Figure 5**).

Inter-reviewer agreement was high for direction coding (Cohen’s κ = 0.95). For the focused risk-of-bias assessment, domain-level agreement ranged from κ = 0.87 to 1.00, with an overall focused risk-of-bias κ of 0.98 (**Supplementary Table 9**). Across all 231 study-cell entries, final direction categories were mixed in 93, positive/harmful in 91, null/no clear association in 15 and not classifiable in 32. Among the 199 classifiable entries, mixed and positive/harmful categories were nearly balanced. In smoking/tobacco × rheumatoid arthritis, mixed results occurred across immune/serologic endpoints (37 of 62 votes), disease occurrence/prevalence (20 of 37) and activity/clinical-course outcomes (20 of 26). Air-pollution × rheumatoid arthritis and air-pollution × systemic lupus erythematosus showed more positive/harmful than mixed votes, whereas occupational × systemic sclerosis showed 17 positive/harmful and 2 mixed votes. These comparisons describe directional heterogeneity across dissimilar outcomes and exposure measures; they are not pooled estimates (**Supplementary Table 17**). Study-cell-level exposure assessment, outcome definition, adjustment variables, effect estimates, direction judgements and focused risk-of-bias assessments are provided in **Supplementary Table 18**. No entry was coded as a robust inverse/protective direction under the prespecified dominant-contrast rules.

The focused risk-of-bias assessment showed that most study-cell entries had some concerns (n = 153), followed by higher risk (n = 40), not assessed (n = 31), lower risk (n = 6) and unclear (n = 1). The not-assessed entries largely reflected records retained for audit but excluded from direction-vote plotting because a cell-specific directional result or risk-of-bias judgement could not be supported reliably. In the harvest plot, each tile represents one study-cell entry, with colour indicating the focused OHAT-style risk-of-bias category. This focused layer was designed to contextualise direction voting in selected high-density cells; it was not a complete risk-of-bias assessment of all 433 studies, a full OHAT certainty assessment, a pooled estimate or a causal summary.

### 3.10 Research-priority ranking across exposure–AIRD combinations

**Figure 6:**
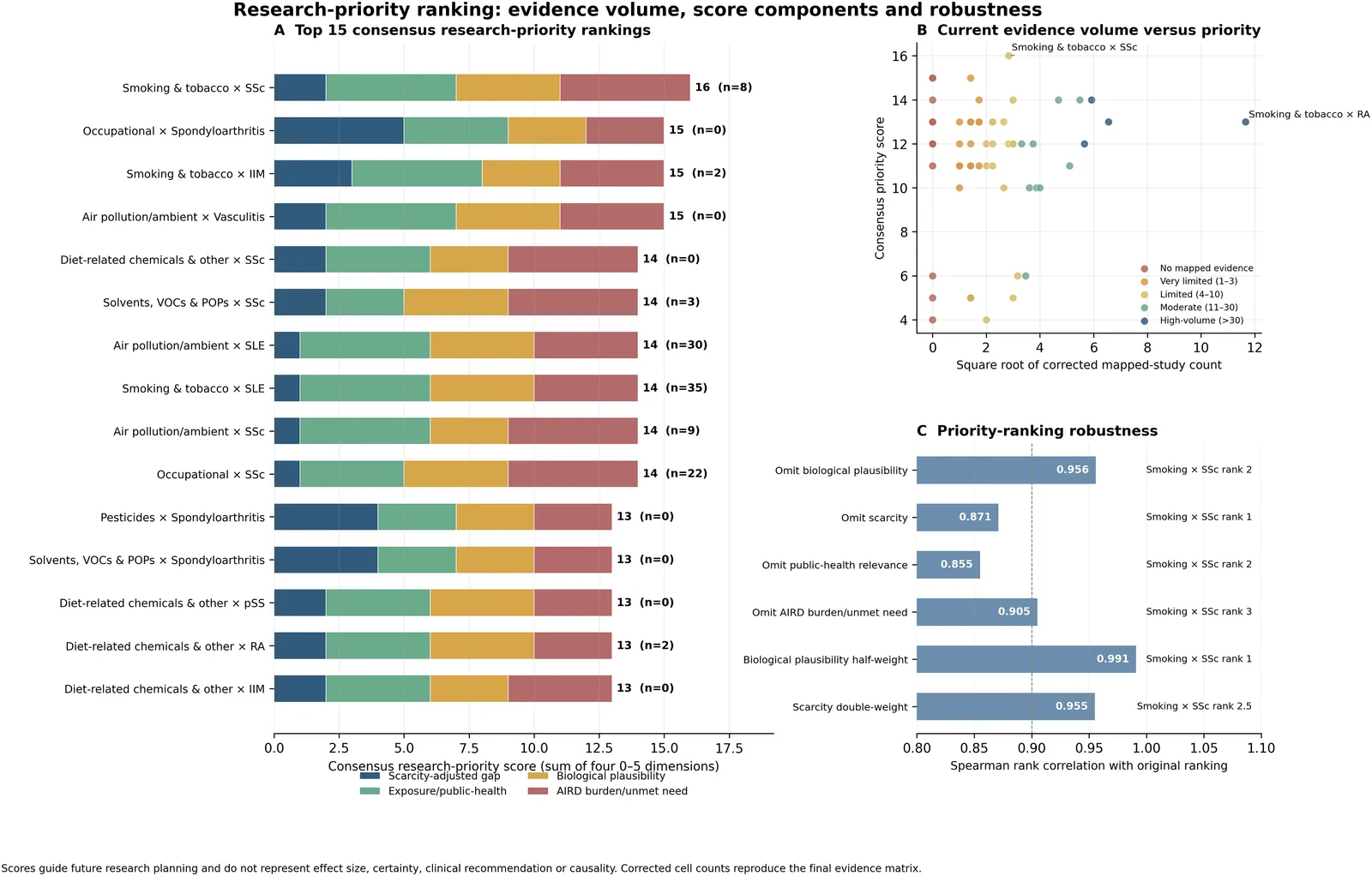
Research-priority ranking across exposure–AIRD combinations. Consensus scores were calculated across 64 exposure-family × AIRD cells using scarcity-adjusted evidence gap, exposure prevalence or public-health relevance, biological plausibility, and AIRD burden or unmet need. **(A)** Top 15 consensus rankings; stacked segments show the four component scores and the annotation reports corrected current evidence volume. **(B)** Corrected evidence volume versus consensus priority across all 64 cells. **(C)** *Post hoc* leave-one-dimension-out and alternative-weight robustness analyses, showing Spearman rank correlation with the original ranking and the corresponding rank of smoking/tobacco × systemic sclerosis. Scores were based on the median of three independent reviewers with adjudication for a spread of at least 2 points (ICC(2,k) = 0.91–0.94). Smoking/tobacco × systemic sclerosis remained within the top three priorities across all alternative schemes, whereas smoking/tobacco × rheumatoid arthritis remained the highest-density evidence cell. Scores support research planning and do not represent effect size, certainty, clinical recommendation or causality.

The research-priority framework was applied across all 64 exposure-family × AIRD cells (**Figure 6**). The top-ranked combinations reflected the joint contribution of scarcity-adjusted evidence gap, exposure or public-health relevance, biological plausibility, and AIRD burden or unmet need. Agreement among the three reviewers was high, with ICC(2,k) values ranging from 0.91 to 0.94 across scoring dimensions and 97–100% of reviewer scores falling within ±1 point. The highest-ranked cell was smoking/tobacco exposure × systemic sclerosis, followed by next-ranked combinations including occupational exposure × spondyloarthritis, smoking/tobacco exposure × idiopathic inflammatory myopathy, and air pollution/ambient exposure × vasculitis. Detailed robustness results are provided in **Supplementary Table 16**.

The priority ranking included both evidence-sparse and higher-volume cells because the score integrated evidence scarcity with public-health relevance, biological plausibility and AIRD burden. Across six alternative weighting or leave-one-dimension-out scenarios, Spearman rank correlations with the original ranking ranged from 0.855 to 0.991 and 9–13 of the original top 15 cells remained in the top 15. Smoking/tobacco × systemic sclerosis ranked between first and third in every scenario. This result does not conflict with smoking/tobacco × rheumatoid arthritis being the highest-density evidence cell: evidence density describes current literature volume, whereas the priority score identifies future research needs by combining scarcity with exposure relevance, plausibility and unmet disease burden. Neither ranking represents association strength or causality. The term “corrected evidence volume” in **Figure 6** refers to the reconciliation of intermediate Other/unspecified counts with the final **Figure 1** 8×8 matrix; it does not denote a change to the 433-study denominator or to the principal non-Other priority cells (**Supplementary Table 16**).

## 4 Discussion

This priority-based evidence map shows that environmental AIRD research is sizeable but uneven, with strong concentration in smoking, air pollution, rheumatoid arthritis and high-income settings. Among 433 included studies, only 104 (24.0%) directly measured an immune/serologic biomarker or mechanistic intermediary, and only 25 (5.8%) measured a pathway-proximal intermediary or omics readout. Most studies therefore contributed exposure–disease, exposure–activity or exposure–phenotype associations rather than direct evidence linking exposure to immune pathways. These findings clarify that the article is mechanism-informed rather than a systematic synthesis of demonstrated mechanisms.

The pattern of mapped evidence is compatible with several partially overlapping mechanistic hypotheses, but the degree of direct human testing differed sharply by exposure–disease pair. Smoking/tobacco × rheumatoid arthritis contained the largest number of immune-informed studies, including analyses of tissue citrullination, DNA methylation, tobacco-derived lipopolysaccharide and shared lung–joint T-cell repertoires (32–35). Even in this cell, however, many studies used serologic subtypes or clinical outcomes rather than measuring pathway intermediates. The evidence should therefore be interpreted as heterogeneous support for targeted mechanistic hypotheses, not as confirmation of a single causal chain.

For systemic lupus erythematosus, most air-pollution studies evaluated disease activity, flares, hospitalisation or other clinical outcomes. A small number directly assessed airway inflammation, serum interleukins or related immune markers (36, 37), but the mapped data did not systematically test NETosis, type I interferon signalling or other proposed pathways across populations. Similarly, smoking-related cytokine studies provide useful examples without establishing a general mechanism (38). For Sjögren’s syndrome, spondyloarthritis, idiopathic inflammatory myopathy and vasculitis, the evidence was too sparse and heterogeneous for firm disease-specific mechanistic interpretation.

**Figure 7:**
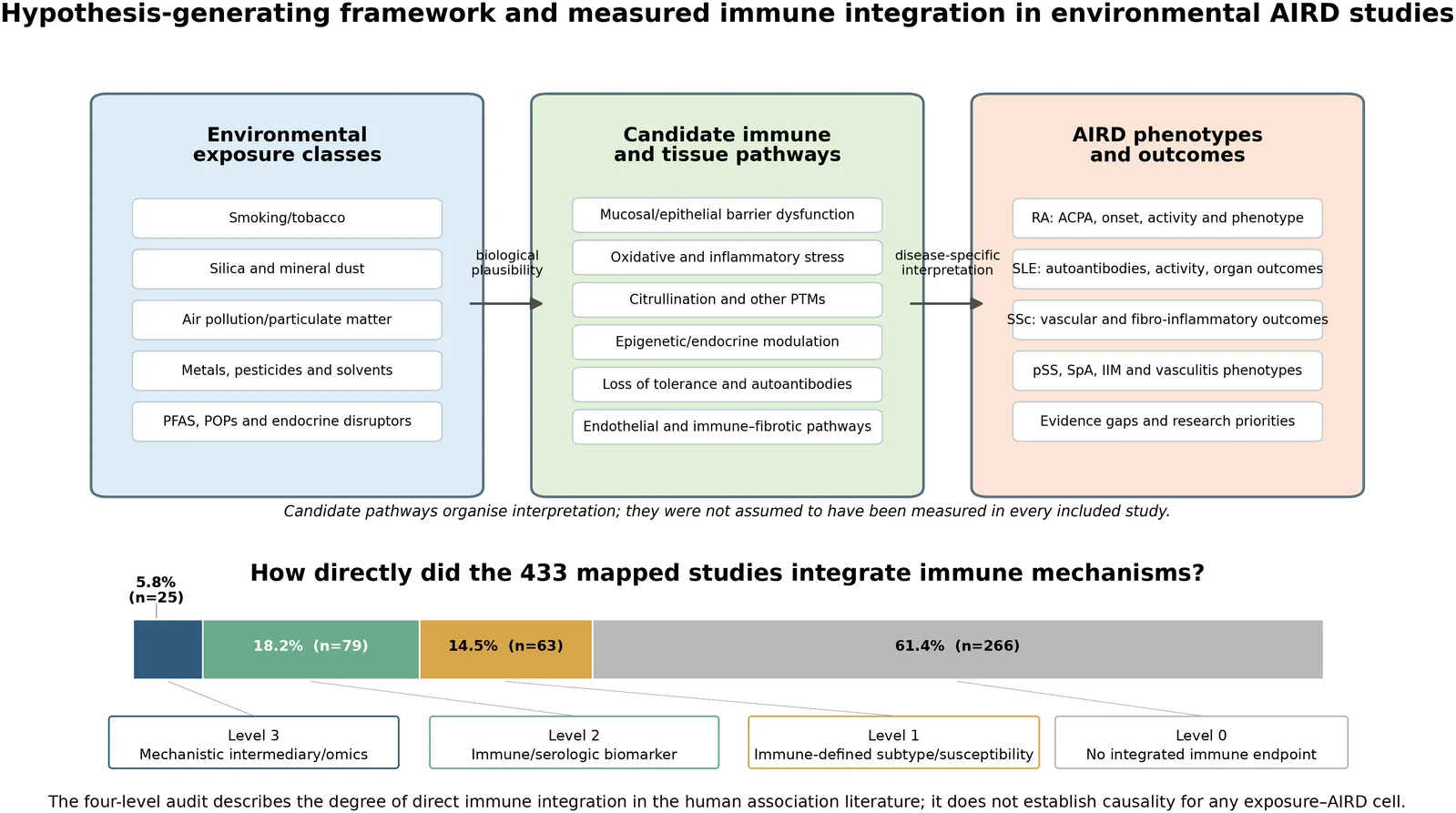
Hypothesis-generating framework and measured immune integration in environmental AIRD studies. The upper framework organises environmental exposure classes, candidate immune or tissue pathways and AIRD phenotypes for biological interpretation. The pathways are hypotheses and are not assumed to have been measured in every study. The lower panel reports the mutually exclusive four-level mechanism-integration audit across all 433 studies: Level 3, direct pathway-proximal intermediary or omics readout (n = 25, 5.8%); Level 2, direct immune/serologic, complement or inflammatory biomarker (n = 79, 18.2%); Level 1, immune-defined subtype or susceptibility analysis (n = 63, 14.5%); and Level 0, no integrated immune endpoint (n = 266, 61.4%). The figure does not imply causality for any exposure–AIRD cell.

This distinction is important for interpreting the map in an immunology context because a high priority score should not be read as evidence that a given exposure causes a given AIRD. Rather, high-priority cells identify where immunological plausibility, exposure relevance, disease burden and evidence scarcity intersect. A conceptual framework linking exposure classes, immune pathways and AIRD phenotypes is provided in **Figure 7** to clarify this hypothesis-generating role and to guide future mechanism-aware epidemiological studies.

The most striking feature of the mapped evidence base is the dominance of smoking and tobacco-related exposures. Nearly half of the priority-coded studies addressed smoking or tobacco exposure, and the largest single evidence cell was smoking/tobacco exposure in rheumatoid arthritis. This pattern is unsurprising given the long-standing interest in smoking as a modifiable environmental factor in rheumatic disease epidemiology, particularly in relation to rheumatoid arthritis onset, autoantibody-positive disease and gene-environment interaction (5, 39–41). However, the scale of this dominance is itself important: it indicates that much of the environmental AIRD literature has been shaped by a traditional risk-factor framework centred on tobacco, rather than by a broader exposome-oriented framework incorporating co-occurring chemical, particulate, occupational and ambient exposures (42).

Air pollution and ambient environmental exposures represented another major but more heterogeneous area, concentrated mainly in rheumatoid arthritis and systemic lupus erythematosus, with fewer studies addressing systemic sclerosis, Sjögren’s syndrome, idiopathic inflammatory myopathy, vasculitis or spondyloarthritis-related diseases (9, 12, 13, 43). The growing presence of ambient air pollution in more recent years suggests that the field is gradually moving from individual lifestyle and occupational risk factors toward population-level environmental determinants (13, 44). This shift is biologically and public-health relevant, because ambient pollution exposures are widespread, socially patterned and potentially modifiable through policy-level interventions (45, 46).

Occupational exposures formed a third important evidence cluster, particularly silica, mineral dust and related inhalational exposures. These patterns are consistent with the long-investigated literature on silica-associated autoimmunity and occupational silica exposure in rheumatic disease (47, 48). In systemic sclerosis, only a minority of mapped studies directly measured immune or molecular endpoints; a recent proteomic study illustrates the type of exposure-linked molecular phenotyping that remains uncommon (49). Most studies relied on job title, occupational history, job-exposure matrices or specific dust and silica measures coupled to clinical outcomes. The relative directional consistency of occupational × systemic sclerosis coexisted with a high proportion of higher-risk entries, so it should not be interpreted as causal confirmation. More standardised exposure matrices, quantitative dose assessment and disease-specific molecular outcomes are needed (50, 51).

Beyond smoking, air pollution and occupational dusts, the evidence base becomes markedly thinner. A small number of studies linked PFAS concentrations to rheumatoid-arthritis immune markers or examined bisphenol A-related methylation signatures in systemic lupus erythematosus (52, 53), but these examples do not support generalisation across chemical classes or AIRDs. Metals, pesticides, solvents, POPs, phthalates and other endocrine-disrupting chemicals were usually represented by small and methodologically diverse sets of studies (10, 11, 14, 15). Mixture-based and biomarker-based approaches remain uncommon, despite real-world co-exposure to correlated chemicals and particles (44, 54).

The disease distribution was similarly uneven. Rheumatoid arthritis accounted for the largest share of studies, followed by systemic lupus erythematosus, systemic sclerosis and primary Sjögren’s syndrome, whereas other AIRDs — including idiopathic inflammatory myopathies, vasculitis, antiphospholipid syndrome, palindromic rheumatism, juvenile idiopathic arthritis and specific spondyloarthritis subtypes — were far less represented. This imbalance means that conclusions about environmental exposures and AIRDs are often driven by rheumatoid arthritis and, to a lesser extent, systemic lupus erythematosus, so generalising across AIRDs is inappropriate unless disease-specific evidence is shown (2–4, 6).

The exposure–disease matrix provides a useful way to identify both mature and underdeveloped areas (21, 22, 26). Smoking–rheumatoid arthritis, smoking–systemic lupus erythematosus, air pollution–rheumatoid arthritis, air pollution–systemic lupus erythematosus and occupational exposure–systemic sclerosis were among the more populated combinations. In contrast, many combinations involving pesticides, PFAS, solvents, endocrine disruptors, metals and less frequently studied AIRDs contained few or no studies. These sparse areas should not be interpreted as evidence of no association; rather, they represent research gaps where the available literature is insufficient to support strong inference (23, 26).

The direction-of-effect synthesis adds an illustrative layer to the map but should not be generalised to all 433 studies (27, 55). Outcome-stratified comparisons showed that the large mixed category in smoking/tobacco × rheumatoid arthritis arose across immune/serologic, disease-occurrence and clinical-course outcomes, rather than from one homogeneous contrast. Air-pollution cells more often showed positive/harmful votes, whereas occupational × systemic sclerosis showed greater directional consistency but substantially higher risk-of-bias concerns. Exposure metrics, windows, outcomes, adjustment sets and populations remained too heterogeneous for a defensible pooled estimate across these broad cells. Direction voting is therefore presented only as a structured description and not as an alternative to effect-size synthesis, certainty grading or causal inference (27, 29, 55).

The research-priority framework extends the map from description toward research planning (25, 26). Its expert-scored dimensions remain partly subjective, despite high inter-reviewer agreement. The new robustness analyses reduce, but do not eliminate, this limitation: rankings remained strongly correlated across alternative schemes, and smoking/tobacco × systemic sclerosis remained within the top three when biological plausibility was removed or down-weighted and when other dimensions were omitted or reweighted. This explains why that cell ranked above smoking/tobacco × rheumatoid arthritis despite having much lower evidence density. The former represents a persistent research need after considering scarcity and unmet burden; the latter represents the most extensively studied current literature. Priority scores should not be used as clinical recommendations or estimates of association strength, effect magnitude or certainty.

The study-design profile suggests that the mapped evidence base remains largely observational. Cohort, case-control, cross-sectional and broadly observational studies dominated, whereas time-series, case-crossover, Mendelian randomisation and exposure-assessment or tool-development studies were uncommon (6, 56). This is understandable because AIRDs are chronic and often relatively uncommon diseases, making prospective environmental studies difficult and expensive; however, the limited use of quasi-experimental, time-series, case-crossover, genetic-instrument or exposure-assessment designs constrains causal interpretation (57–59). Future work should address time-varying exposure, latency, selection bias, reverse causation and residual confounding with designs and analytic strategies appropriate to the exposure and disease process (60, 61).

The methodological-signal distribution reinforces this conclusion. Interaction analyses were relatively common, reflecting interest in effect modification and gene–environment or exposure–phenotype heterogeneity, but sensitivity analyses, nonlinear modelling, complex survey weighting and mixture methods were much less frequent. Although many studies were coded as addressing more than one exposure domain or agent, only a small minority applied formal mixture-method analysis — broad multi-agent coverage in an evidence map does not mean that studies jointly modelled correlated co-exposures (62). Future studies should more often incorporate multipollutant models, weighted quantile sum regression, Bayesian kernel machine regression, quantile g-computation, exposome-wide approaches or other mixture-oriented frameworks when data structure permits (63–66).

The geographic distribution reveals a further limitation of the evidence base. The United States contributed the largest number of studies, followed by several European countries and China, whereas many regions with high environmental exposure burdens were minimally represented or absent (45, 46). This imbalance may affect more than external validity: pesticide formulations, occupational dust composition, pollution mixtures, exposure intensity, co-exposures, genetic background, microbiome profiles, healthcare access and diagnostic pathways can vary across regions (4, 67, 68). Consequently, a pathway or exposure metric studied in one setting may not transfer directly to rapidly industrialising or underrepresented regions. Occupational silica, ambient pollution mixtures and pesticide-related priority cells are particularly sensitive to this context.

The temporal pattern indicates that the field has expanded over time, particularly in recent years, likely reflecting increasing recognition of environmental determinants of immune-mediated diseases, improved exposure modelling, larger electronic health datasets and greater interest in air pollution and exposome research (4, 44, 69, 70). Nonetheless, publication growth is descriptive and does not necessarily imply methodological maturity.

The source-journal distribution and supplementary author-keyword display provide contextual evidence of a multidisciplinary field spanning rheumatology, environmental health, epidemiology, toxicology and public health. These descriptors were not used to determine evidence density or research priorities. The cross-disciplinary spread is a strength because environmental AIRD research requires clinical disease expertise, exposure science, epidemiology, biostatistics and public-health interpretation (4, 44). It also creates a practical challenge: terminology, exposure classification, outcome definitions and reporting standards differ between rheumatology and environmental health audiences, making harmonisation essential before the field can move toward comparable evidence synthesis and causal interpretation (56).

This study has several strengths relevant to environmental immunology evidence synthesis. First, it used two major bibliographic databases and documented a large corpus from search through deduplication, screening, prioritisation, eligibility assessment and figure generation (71, 72). Second, the workflow explicitly separated title/abstract exclusions, retained-but-deferred records, reports not retrieved and full-text exclusions, avoiding the common problem of treating all non-included records as equivalent. Third, the final map was based on record-level priority coding and eligibility verification, rather than title-only or abstract-only publication counts (22). Fourth, non-mutually exclusive exposure and disease coding allowed studies addressing multiple exposures or AIRDs to be represented more accurately. Fifth, dense-cell direction-of-effect synthesis and focused risk-of-bias assessment provided context for the most populated evidence cells without overstating causal inference (27). Finally, search strategies, audit materials, coding outputs, harmonisation dictionaries, plot-ready data, scoring materials and software information were deposited in an open repository, improving transparency and reproducibility (73).

Several limitations should be acknowledged. First, this was a priority-based evidence map rather than an exhaustive full-text systematic review of all 7,725 retained records; eligible studies may remain in the deferred branch (22, 23); no full-text re-review or random-sample audit of the 5,093 deferred records was conducted, so the magnitude and direction of any resulting underrepresentation, particularly in sparse exposure–AIRD cells, cannot be quantified. Second, some retained records had limited full-text auditability for selected descriptive fields, and residual misclassification remains possible. Third, exposure and AIRD categories were non-mutually exclusive. Fourth, direction-of-effect synthesis and focused risk-of-bias assessment were limited to four selected cells and are illustrative rather than representative of the full map (27, 29). Fifth, no pooled estimates were calculated because the broad cells combined materially different exposures, windows, outcomes, designs and adjustment strategies; disease- or exposure-specific meta-analyses may still be appropriate when sufficiently comparable studies are identified. Sixth, the research-priority framework involved expert judgement despite high agreement and supportive robustness analyses (26, 74). Seventh, the mechanism-integration audit was *post hoc* and classified the degree of exposure-linked immune measurement rather than the validity or causal relevance of an individual biomarker. It was not a *de novo* mechanistic risk-of-bias assessment.

Additional limitations relate to data sources and reporting. The review was based on the Web of Science Core Collection and Scopus, so eligible biomedical studies indexed only in MEDLINE/PubMed, Embase, regional databases or non-English sources may have been missed; findings should be interpreted as a Web of Science Core Collection/Scopus-based priority-coded map rather than a fully exhaustive biomedical systematic review (75, 76). English-language filtering may have introduced language bias and reduced representation from non-English-speaking regions. Search and indexing metadata can also affect publication-year assignment, document-type classification and keyword availability. Country information was missing in a substantial number of studies, limiting geographic interpretation. The supplementary author-keyword display was restricted to studies with available keywords and depended on inconsistent author-supplied terminology; it was not used in the main evidence-gap or priority analyses. Finally, display-level harmonisation was necessary for interpretable figures, but harmonised categories may still mask differences in exposure measurement, intensity, timing, route and biological relevance (60). Future disease- or exposure-specific systematic reviews should therefore include MEDLINE/PubMed, Embase and relevant regional databases when the question requires exhaustive biomedical retrieval.

For environmental immunology and autoimmune-disease research, the map highlights five practical priorities. First, exposure assessment should move beyond ever/never or self-reported exposure variables toward time-windowed, quantitative, geocoded, biomonitoring-based or job-exposure-matrix approaches where feasible. Second, studies should more often account for co-exposures and mixtures, because real-world populations encounter correlated chemical, particulate, occupational and ambient exposures rather than isolated agents. Third, AIRD outcomes should be phenotyped more granularly, including autoantibody status, disease activity, organ involvement, clinical subtype and preclinical disease states. Fourth, future work should incorporate immune biomarkers or mechanistic intermediates when possible, including markers of inflammation, oxidative stress, epithelial-barrier injury, epigenetic regulation, autoantibody formation and disease-specific immune signatures. Fifth, future studies should include more diverse populations and underrepresented regions so that evidence reflects variation in exposure profiles, occupational conditions, healthcare access, genetic background and diagnostic pathways. Cell-specific systematic reviews or meta-analyses may be warranted where evidence volume and methodological comparability are sufficient, whereas sparse but high-priority cells should be addressed through new mechanism-aware epidemiological studies (26).

## 5 Conclusions

This priority-based evidence map identified 433 studies addressing environmental exposures and AIRDs. The literature was dominated by smoking/tobacco, rheumatoid arthritis and studies from North America and Europe, whereas many chemical exposures, AIRDs and regions were sparsely represented. The *post hoc* audit showed that 24.0% of studies directly measured an immune/serologic biomarker or mechanistic intermediary and only 5.8% measured a pathway-proximal intermediary or omics readout. Dense-cell direction patterns were heterogeneous, and risk-of-bias concerns limited interpretation. Priority rankings were broadly robust to alternative scoring schemes but remain research-planning tools rather than evidence of association strength or causality. Future work should jointly measure quantitative exposures, time windows, mixtures, immune intermediates and granular AIRD phenotypes in geographically diverse populations.

## Data Availability

The processed study-level data and analytical materials supporting this study are available in Zenodo at https://doi.org/10.5281/zenodo.21438501. The repository includes study-level coding and audit materials, harmonisation resources, mechanism-integration and priority-robustness analyses, and figure-ready outputs. No copyrighted full-text articles or raw bulk database exports from the Web of Science Core Collection or Scopus are redistributed.
