# Supplementary material for: Environmental exposures in autoimmune inflammatory rheumatic diseases: a priority-based evidence map, immune-mechanism integration audit and research agenda
Source: Front Immunol. 2026 Aug 10;17:1916679. doi: 10.3389/fimmu.2026.1916679 (PMC13500649; doi:10.3389/fimmu.2026.1916679)
Supplement: Supplementary file 1 [file Table6.docx]

**Supplementary File 2. Adapted PRISMA-ScR checklist for the priority-based mapping review**

*Environmental exposures in autoimmune inflammatory rheumatic diseases: a priority-based evidence map, immune-mechanism integration audit and research agenda*

Reporting guideline basis: Preferred Reporting Items for Systematic reviews and Meta-Analyses extension for Scoping Reviews (PRISMA-ScR). This adapted checklist maps PRISMA-ScR items to the revised manuscript, supplementary materials, retrospective OSF registration and Zenodo repository. The article is a mechanism-informed, priority-based evidence map rather than an exhaustive full-text scoping review of every retained record. The revision adds a clearly labelled post hoc audit of immune-mechanism integration, dense-cell stratification and research-priority robustness analyses. The checklist is therefore used as an adapted transparency aid rather than as a claim of conventional full-scope scoping-review compliance.

| Section | Topic | Item | PRISMA-ScR checklist item | Reported | Location / notes |
| --- | --- | --- | --- | --- | --- |
| TITLE | Title | 1 | Identify the report as a scoping review. | Yes, with qualification | Title page / manuscript title. The revised title identifies the report as a priority-based evidence map with an immune-mechanism integration audit and research agenda rather than as a conventional exhaustive scoping review. |
| ABSTRACT | Structured summary | 2 | Provide a structured summary that includes, as applicable, background, objectives, eligibility criteria, sources of evidence, charting methods, results and conclusions that relate to the review questions and objectives. | Yes | Revised Abstract. The journal-formatted abstract remains a single paragraph and reports the rationale, eligibility, sources, focused mapping approach, post hoc mechanism-integration audit, principal findings, ranking robustness and conclusions. |
| INTRODUCTION | Rationale | 3 | Describe the rationale for the review in the context of what is already known. Explain why the review questions/objectives lend themselves to a scoping review approach. | Yes | Introduction, including the rationale for a broad evidence map across environmental exposure families and AIRD phenotypes. |
| INTRODUCTION | Objectives | 4 | Provide an explicit statement of the questions and objectives being addressed with reference to their key elements, such as population or participants, concepts and context, or other relevant key elements used to conceptualize the review questions and/or objectives. | Yes | Introduction / objective statement. The revised objectives distinguish evidence structure, directionality, degree of direct immune-endpoint integration and future research priority. |
| METHODS | Protocol and registration | 5 | Indicate whether a review protocol exists; state if and where it can be accessed, and if available, provide registration information, including the registration number. | Yes | Methods 2.1 and data availability materials. A retrospective OSF registration documents the final research question, eligibility criteria, search strategy, prioritisation framework and analysis plan: doi:10.17605/OSF.IO/UVX93. Versioned protocol and audit materials are available in Zenodo. |
| METHODS | Eligibility criteria | 6 | Specify characteristics of the sources of evidence used as eligibility criteria, such as years considered, language and publication status, and provide a rationale. | Yes | Methods 2.4 and Supplementary Methods. Eligibility criteria define original human environmental exposure–AIRD studies, publication years, language restrictions, exposure scope and exclusion rules. |
| METHODS | Information sources | 7 | Describe all information sources in the search, such as databases with dates of coverage and contact with authors to identify additional sources, as well as the date the most recent search was executed. | Yes | Methods 2.2 and Supplementary Methods. Web of Science Core Collection and Scopus were searched and exported on 18 May 2026; complete database details are documented in the supplementary and repository materials. |
| METHODS | Search | 8 | Present the full electronic search strategy for at least one database, including any limits used, such that it could be repeated. | Yes | Supplementary Methods and OSF/Zenodo materials. Full electronic search strings, filters, field tags, export counts and sensitivity-search documentation are provided. |
| METHODS | Selection of sources of evidence | 9 | State the process for selecting sources of evidence, including screening and eligibility, included in the scoping review. | Yes | Methods 2.5–2.8; Fig. 1. The workflow reports title/abstract screening, focused-priority selection, deferred records, full-text retrieval, eligibility assessment, exclusion reasons and final dataset freeze. |
| METHODS | Data charting process | 10 | Describe the methods of charting data from the included sources of evidence, such as calibrated forms or forms tested by the team before use, whether data charting was done independently or in duplicate, and any processes for obtaining and confirming data from investigators. | Yes | Methods 2.9–2.10 and Supplementary Methods. Data were extracted into structured templates; calibration, reviewer verification, harmonisation, source-status recording and audit procedures are described. |
| METHODS | Data items | 11 | List and define all variables for which data were sought and any assumptions and simplifications made. | Yes | Methods 2.9–2.14; Table 2; Supplementary Tables S14–S17 and record-level repository files. Variables include bibliographic and epidemiological fields, exposure/AIRD classifications, direction and focused risk-of-bias fields, priority components, the four-level immune-integration hierarchy, outcome strata and robustness scenarios. |
| METHODS | Critical appraisal of individual sources of evidence | 12 | If done, provide a rationale for conducting a critical appraisal of included sources of evidence; describe the methods used and how this information was used in any data synthesis, if appropriate. | Yes, with qualification | Methods 2.12. A focused OHAT-style risk-of-bias assessment was conducted only for four selected high-density exposure–AIRD cells, not for all 433 included studies. This layer contextualised dense-cell direction-of-effect synthesis and was not used to generate pooled estimates or certainty grades. |
| METHODS | Synthesis of results | 13 | Describe the methods of handling and summarizing the data that were charted. | Yes | Methods 2.11–2.16. The revised synthesis includes descriptive evidence mapping, dense-cell SWiM-aligned direction synthesis, focused risk-of-bias contextualisation, 64-cell research-priority scoring, a post hoc four-level immune-mechanism integration audit, dense-cell stratification and alternative-ranking analyses. |
| RESULTS | Selection of sources of evidence | 14 | Give numbers of sources of evidence screened, assessed for eligibility and included in the review, with reasons for exclusions at each stage, ideally using a flow diagram. | Yes | Results 3.1; Fig. 1; Supplementary Table S3. The manuscript reports 17,089 records before deduplication, 12,497 screened records, 7,725 retained in the broader screening pool, 2,632 focused-priority reports, 2,629 assessed reports and 433 included studies. |
| RESULTS | Characteristics of sources of evidence | 15 | For each source of evidence, present characteristics for which data were charted and provide the citations. | Yes | Results 3.2–3.10; Tables 1–2; Supplementary Tables S1–S17 and record-level repository materials. Study characteristics, immune-integration levels, dense-cell outcome strata and priority-score robustness are reported. |
| RESULTS | Critical appraisal within sources of evidence | 16 | If done, present data on critical appraisal of included sources of evidence. | Yes, with qualification | Results 3.9; Fig. 5; Supplementary Tables S8–S9 and the record-level dense-cell spreadsheet. Focused risk-of-bias results apply only to four selected cells and are not presented as a map-wide certainty assessment. |
| RESULTS | Results of individual sources of evidence | 17 | For each included source of evidence, present the relevant data that were charted and that relate to the review questions and objectives. | Yes | Tables 1–2, Supplementary Table S1 spreadsheet and record-level Zenodo files provide study-level characteristics and coding. The dense-cell spreadsheet additionally documents exposure metrics, outcome definitions, selected models, supporting results, direction and focused risk-of-bias decisions. |
| RESULTS | Synthesis of results | 18 | Summarize and/or present the charting results as they relate to the review questions and objectives. | Yes | Results 3.2–3.10. The revised manuscript reports exposure and AIRD distributions, the evidence map, four-level immune-mechanism integration, methodological/geographic descriptors, dense-cell direction and risk-of-bias results, and research-priority robustness. |
| DISCUSSION | Summary of evidence | 19 | Summarize the main results, including an overview of concepts, themes and types of evidence available; link to the review questions and objectives; and consider the relevance to key groups. | Yes | Discussion. The revised discussion distinguishes exposure–disease association evidence from directly measured immune biomarkers or pathway-proximal intermediates, compares mechanistic integration across exposure–AIRD pairs and interprets ranking robustness cautiously. |
| DISCUSSION | Limitations | 20 | Discuss the limitations of the scoping review process. | Yes | Discussion / limitations. Limitations include the priority-based rather than exhaustive design, deferred records, database and language restrictions, limited auditability, non-mutually exclusive coding, selected-cell risk-of-bias scope, the post hoc nature of the mechanism-integration audit and expert judgement in priority scoring despite robustness analyses. |
| DISCUSSION | Conclusions | 21 | Provide a general interpretation of the results with respect to the review questions and objectives, as well as potential implications and/or next steps. | Yes | Revised Conclusions and final Discussion priorities. The manuscript interprets the map as an association-focused evidence base with limited direct mechanism integration and a transparent, sensitivity-tested research agenda. |
| FUNDING | Funding | 22 | Describe sources of funding for the included sources of evidence, as well as sources of funding for the scoping review. Describe the role of the funders of the scoping review. | Yes, with qualification | Funding statement. Funding sources for the present review are reported. Funding sources for each included source were not systematically analysed because this was not an objective of the priority-based evidence map. |

**Abbreviations:** AIRD = autoimmune inflammatory rheumatic disease; DOI = digital object identifier; OSF = Open Science Framework; PRISMA-ScR = Preferred Reporting Items for Systematic reviews and Meta-Analyses extension for Scoping Reviews; RoB = risk of bias; SWiM = Synthesis Without Meta-analysis.

**Guideline reference:** Tricco AC, Lillie E, Zarin W, O'Brien KK, Colquhoun H, Levac D, et al. PRISMA Extension for Scoping Reviews (PRISMA-ScR): Checklist and Explanation. Ann Intern Med (2018) 169:467-473. doi:10.7326/M18-0850.
