# Supplementary material for: Environmental exposures in autoimmune inflammatory rheumatic diseases: a priority-based evidence map, immune-mechanism integration audit and research agenda
Source: Front Immunol. 2026 Aug 10;17:1916679. doi: 10.3389/fimmu.2026.1916679 (PMC13500649; doi:10.3389/fimmu.2026.1916679)
Supplement: Supplementary file 2 [file Table7.docx]

**Supplementary Materials**

Environmental exposures in autoimmune inflammatory rheumatic diseases: a priority-based evidence map, immune-mechanism integration audit and research agenda

All distributions use the frozen main-analysis denominator of n = 433 priority-coded studies unless otherwise stated. Categories are not mutually exclusive where indicated. Primary figures and tables use this main-analysis set; a restricted-auditability sensitivity frame (n = 292) excludes 141 retained records with limited full-text auditability and metadata-assisted descriptive coding. The revised supplementary materials additionally document a post hoc four-level audit of immune-mechanism integration, outcome-stratified comparisons within the four dense cells and robustness analyses of the 64-cell research-priority ranking. These nested analyses delimit the scope of inference and do not convert the map into an exhaustive systematic review or a causal synthesis. Full record-level audit tables, adjudication records and sensitivity matrices are provided in the accompanying spreadsheets and the updated Zenodo repository version.

Mechanism-integration interpretation note: the audit classifies the degree to which an included human study integrated an exposure-linked immune endpoint. Level 3 denotes a pathway-proximal mechanistic intermediary or omics readout; Level 2 an immune, serologic, complement or inflammatory biomarker; Level 1 an immune-defined disease subtype or genetic susceptibility/interaction without a measured intermediary; and Level 0 no integrated immune endpoint. These categories describe what was measured and do not establish that an exposure caused an AIRD or a specific immune pathway. Biological plausibility remained a transparent research-priority dimension rather than evidence of causality.

# **Supplementary Methods**

## Search and export documentation

The Web of Science Core Collection and Scopus were searched and exported on 18 May 2026. Searches combined AIRD terms with environmental exposure terms and were limited to English-language article or review records in the intended publication window of 2005–2025. Review records were exported for search sensitivity, audit and reference-tracking purposes but were excluded during eligibility assessment if they were non-original publications. Records were retrieved under a 2005–2025 publication-window filter applied at database export (18 May 2026). Two included records were available online (early access) and indexed within this window but were subsequently assigned a 2026 final issue/citation year by the publisher; both were retained, as their online availability and indexing fell within the search window. This does not affect any count, which is based on the frozen set of 433 included studies.

The Web of Science Core Collection export contained 5,173 English-language article/review records after final filtering, and the Scopus export contained 11,916 records after final filtering. The exact final database queries, filters, export counts and exported field groups are provided below so that the search can be reproduced without relying only on the repository files. For readability, repeated field-specific term blocks are displayed below as compact Boolean logic; the deposited query files retain the complete copy-paste database strings used for audit.

**Supplementary search strings and export manifest summary**

**Web of Science Core Collection final search string (searched in title, abstract and author-keyword fields; final filters: Article OR Review Article; English; publication date 2005-01-01 to 2025-12-31):**

DiseaseTerms = ("rheumatoid arthritis" OR "systemic lupus erythematosus" OR SLE OR "lupus nephritis" OR "sjogren* syndrome" OR "primary sjogren*" OR "systemic sclerosis" OR "idiopathic inflammatory myopath*" OR "inflammatory myopath*" OR "inclusion body myositis" OR dermatomyositis OR polymyositis OR "ANCA-associated vasculitis" OR "antiphospholipid syndrome" OR "axial spondyloarthritis" OR "ankylosing spondylitis" OR "psoriatic arthritis" OR "mixed connective tissue disease" OR "undifferentiated connective tissue disease" OR "adult-onset Still* disease" OR AOSD OR "autoimmune rheumatic disease*" OR "systemic autoimmune rheumatic disease*")
ExposureTerms = ("air pollution" OR particulate* OR "fine particulate matter" OR PM2.5 OR PM10 OR ozone OR "nitrogen dioxide" OR NO2 OR "sulfur dioxide" OR SO2 OR "traffic-related air pollution" OR "indoor air" OR "indoor air pollution" OR smoking OR tobacco OR "cigarette smoke" OR "secondhand smoke" OR "passive smoking" OR silica OR asbestos OR "dust exposure" OR "organic dust" OR "inorganic dust" OR pesticide* OR herbicide* OR insecticide* OR "solvent exposure*" OR "organic solvent*" OR "volatile organic compound*" OR VOC* OR benzene OR formaldehyde OR trichloroethylene OR "polycyclic aromatic hydrocarbon*" OR PAH* OR "heavy metal*" OR "lead exposure" OR "blood lead" OR "urinary lead" OR "lead level*" OR cadmium OR mercury OR arsenic OR chromium OR nickel OR manganese OR phthalate* OR bisphenol* OR PFAS OR PFOA OR PFOS OR "per- and polyfluoroalkyl substances" OR "polychlorinated biphenyl*" OR PCB* OR dioxin* OR "endocrine disrupt*" OR "occupational exposure*" OR "chemical mixture*" OR "mixed exposure*" OR multipollut* OR "multi-pollutant" OR microplastic* OR nanoplastic* OR exposom*)
Final WoSCC logic = (TI=(DiseaseTerms) OR AB=(DiseaseTerms) OR AK=(DiseaseTerms)) AND (TI=(ExposureTerms) OR AB=(ExposureTerms) OR AK=(ExposureTerms))
Final filters = Article OR Review Article; English; publication date 2005-01-01 to 2025-12-31.

Web of Science Core Collection export summary: final refined result count = 5,173; Article/Review before English filter = 5,276; Article/Review + English final frozen set = 5,173. Tab-delimited records were exported in six batches of up to 1,000 records, and cited references were exported in 11 batches of up to 500 records.

**Scopus final search string (TITLE-ABS-KEY; final filters: Article OR Review; English; PUBYEAR > 2004 AND PUBYEAR < 2026):**

TITLE-ABS-KEY("rheumatoid arthritis" OR "systemic lupus erythematosus" OR SLE OR "lupus nephritis" OR "sjogren* syndrome" OR "primary sjogren*" OR "systemic sclerosis" OR "idiopathic inflammatory myopath*" OR "inflammatory myopath*" OR "inclusion body myositis" OR dermatomyositis OR polymyositis OR "ANCA-associated vasculitis" OR "antiphospholipid syndrome" OR "axial spondyloarthritis" OR "ankylosing spondylitis" OR "psoriatic arthritis" OR "mixed connective tissue disease" OR "undifferentiated connective tissue disease" OR "adult-onset Still* disease" OR AOSD OR "autoimmune rheumatic disease*" OR "systemic autoimmune rheumatic disease*") AND TITLE-ABS-KEY("air pollution" OR particulate* OR "fine particulate matter" OR PM2.5 OR PM10 OR ozone OR "nitrogen dioxide" OR NO2 OR "sulfur dioxide" OR SO2 OR "traffic-related air pollution" OR "indoor air" OR "indoor air pollution" OR smoking OR tobacco OR "cigarette smoke" OR "secondhand smoke" OR "passive smoking" OR silica OR asbestos OR "dust exposure" OR "organic dust" OR "inorganic dust" OR pesticide* OR herbicide* OR insecticide* OR "solvent exposure*" OR "organic solvent*" OR "volatile organic compound*" OR VOC* OR benzene OR formaldehyde OR trichloroethylene OR "polycyclic aromatic hydrocarbon*" OR PAH* OR "heavy metal*" OR "lead exposure" OR "blood lead" OR "urinary lead" OR "lead level*" OR cadmium OR mercury OR arsenic OR chromium OR nickel OR manganese OR phthalate* OR bisphenol* OR PFAS OR PFOA OR PFOS OR "per- and polyfluoroalkyl substances" OR "polychlorinated biphenyl*" OR PCB* OR dioxin* OR "endocrine disrupt*" OR "occupational exposure*" OR "chemical mixture*" OR "mixed exposure*" OR multipollut* OR "multi-pollutant" OR microplastic* OR nanoplastic* OR exposom*) AND NOT TITLE-ABS-KEY("fish consumption" OR seafood OR aquaculture OR makardhwaja OR "gold containing" OR "mud pool" OR phytoremediation OR mycorrhiz* OR ectomycorrhiz* OR fungi OR fungal OR rat OR rats OR mouse OR mice OR murine OR zebrafish) AND PUBYEAR > 2004 AND PUBYEAR < 2026
Final filters = Article OR Review; English.

Scopus export summary: final filtered set = 11,916 records. Exported field groups included citation information, bibliographical information, abstract and keywords, and funding details. References/cited references were not exported from Scopus because citation-network analyses were not conducted in the final workflow; the supplementary author-keyword display is included as contextual description only.

Additional sensitivity searches were conducted for selected boundary terms, including trace metal biomarker terms and the scleroderma/systemic-sclerosis term expansion. Their exact queries and export manifests are deposited with the audit materials and were used for QC rather than to redefine the main frozen corpus.

Deduplication followed a hierarchical rule set. Records were first matched using DOI exact matching when DOI values were present. Records without usable DOI matches were compared using normalised title strings after lower-casing, punctuation removal, whitespace standardisation and removal of common database artefacts. Potential residual duplicates were further checked using combinations of first author, publication year, source title and record title. High-confidence duplicates were removed automatically; ambiguous pairs were retained in the corpus and carried forward for audit rather than deleted manually.

## Supplementary sensitivity search documentation

Two supplementary Web of Science Core Collection sensitivity searches were archived to check boundary coverage for trace-metal terminology and systemic-sclerosis/scleroderma terminology. These searches were not used to change the frozen main-analysis denominator but were retained as audit materials for search sensitivity. Archived screenshots documented final sensitivity result counts of 25 records for the trace-metal search and 1,150 records for the scleroderma search. Export batches were recorded as follows:

**WoSCC sensitivity export manifest

1) sensitivity_scleroderma
citedrefs_txt:
woscc_sensitivity_scleroderma_citedrefs_part01.txt to part03.txt

tabdelim_txt:
woscc_sensitivity_scleroderma_tabdelim_part01.txt to part02.txt

2) sensitivity_trace_metals
citedrefs_txt:
woscc_sensitivity_trace_metals_citedrefs_part01.txt

tabdelim_txt:
woscc_sensitivity_trace_metals_tabdelim_part01.txt**

**WoSCC sensitivity query 1: copper/zinc/selenium trace-metal terms**

DiseaseTerms = ("rheumatoid arthritis" OR "systemic lupus erythematosus" OR SLE OR "lupus nephritis" OR "sjogren* syndrome" OR "primary sjogren*" OR "systemic sclerosis" OR "idiopathic inflammatory myopath*" OR "inflammatory myopath*" OR "inclusion body myositis" OR dermatomyositis OR polymyositis OR "ANCA-associated vasculitis" OR "antiphospholipid syndrome" OR "axial spondyloarthritis" OR "ankylosing spondylitis" OR "psoriatic arthritis" OR "mixed connective tissue disease" OR "undifferentiated connective tissue disease" OR "adult-onset Still* disease" OR AOSD OR "autoimmune rheumatic disease*" OR "systemic autoimmune rheumatic disease*")
TraceMetalTerms = ("blood copper" OR "serum copper" OR "urinary copper" OR "copper exposure" OR "blood zinc" OR "serum zinc" OR "urinary zinc" OR "zinc exposure" OR "blood selenium" OR "serum selenium" OR "urinary selenium" OR "selenium exposure")
WoSCC sensitivity logic = (TI=(DiseaseTerms) OR AB=(DiseaseTerms) OR AK=(DiseaseTerms)) AND (TI=(TraceMetalTerms) OR AB=(TraceMetalTerms) OR AK=(TraceMetalTerms))
Final filters = Article OR Meeting Abstract; English; publication date 2005-01-01 to 2025-12-31.

**WoSCC sensitivity query 2: systemic sclerosis/scleroderma boundary search**

SScTerms = ("systemic sclerosis" OR (scleroderma NOT (fung* OR mycorrhiz* OR phytoremediation OR pine* OR plant* OR ectomycorrhiz*)))
ExposureTerms = ("air pollution" OR particulate* OR "fine particulate matter" OR PM2.5 OR PM10 OR ozone OR "nitrogen dioxide" OR NO2 OR "sulfur dioxide" OR SO2 OR "traffic-related air pollution" OR "indoor air" OR "indoor air pollution" OR smoking OR tobacco OR "cigarette smoke" OR "secondhand smoke" OR "passive smoking" OR silica OR asbestos OR "dust exposure" OR "organic dust" OR "inorganic dust" OR pesticide* OR herbicide* OR insecticide* OR "solvent exposure*" OR "organic solvent*" OR "volatile organic compound*" OR VOC* OR benzene OR formaldehyde OR trichloroethylene OR "polycyclic aromatic hydrocarbon*" OR PAH* OR "heavy metal*" OR "lead exposure" OR "blood lead" OR "urinary lead" OR "lead level*" OR cadmium OR mercury OR arsenic OR chromium OR nickel OR manganese OR phthalate* OR bisphenol* OR PFAS OR PFOA OR PFOS OR "per- and polyfluoroalkyl substances" OR "polychlorinated biphenyl*" OR PCB* OR dioxin* OR "endocrine disrupt*" OR "occupational exposure*" OR "chemical mixture*" OR "mixed exposure*" OR multipollut* OR "multi-pollutant" OR microplastic* OR nanoplastic* OR exposom*)
WoSCC sensitivity logic = (TI=(SScTerms) OR AB=(SScTerms) OR AK=("systemic sclerosis" OR scleroderma)) AND (TI=(ExposureTerms) OR AB=(ExposureTerms) OR AK=(ExposureTerms))
Final filters = Article OR Review Article; English; publication date 2005-01-01 to 2025-12-31.

These sensitivity-search outputs were used as audit checks only; final inclusion remained governed by the predefined eligibility criteria and the final priority-coded analytic dataset.

## Focused screening and coding structure

The deduplicated corpus contained 12,497 records. Title/abstract screening excluded 4,772 records and retained 7,725 records in the broader screening pool. Title/abstract screening was performed independently by two reviewers after pilot calibration, with disagreements resolved by consensus and senior adjudication when needed. Operationally, a direct exposure–AIRD link required an eligible environmental exposure and an eligible AIRD population, outcome or phenotype within the same human study, with a design likely to permit extraction of the exposure–AIRD contrast. Indirect or adjacent records included mechanistic studies without an eligible human AIRD population or outcome, AIRD studies in which exposure was only a covariate or background descriptor, and exposure-focused studies without an extractable AIRD measure. The broader pool was stratified into a focused priority set (2,632), a secondary deferred set (3,669) and an additional deferred set retained for later specialised review (1,424). Deferred records were retained in the search trail and flow diagram but were not treated as formal exclusions.

Full texts were sought for all 2,632 focused-priority records. Three reports were not retrieved. Among 2,629 assessed reports, 2,196 were excluded after full-text assessment and 433 were retained in the final priority-coded evidence map. Eligibility assessment and exclusion-reason coding used a primary reviewer plus second-reviewer verification workflow: all retained studies, all full-text-unavailable records, all boundary-sensitive records and all records with uncertain or overlapping exposure/outcome classification were independently checked by a second reviewer. Disagreements were resolved by consensus before the final analytic dataset was frozen.

## Boundary, harmonisation and sensitivity framework

Eligibility was restricted to original human studies linking target environmental exposures with AIRD populations, outcomes, phenotypes, clinical features, serological features or disease-related measures. Environmental exposures were defined as chemical, particulate, occupational, tobacco-related or related environmental contaminant exposures. Drug exposure, treatment-response studies, non-human studies, mechanistic-only studies and general social, lifestyle or dietary variables without a target environmental exposure were excluded.

Display-term harmonisation was performed to improve interpretability of figures and tables while preserving raw record-level coding. Display-only merges and label refinements did not change inclusion decisions, denominators or underlying coding. The main analysis used all 433 retained studies. Sensitivity denominator 1 excluded 11 authoritative boundary records (n = 422); sensitivity denominator 2 excluded 141 retained records with limited full-text auditability and metadata-assisted descriptive coding (n = 292); the combined sensitivity denominator was n = 281 because the boundary and restricted-auditability lists had zero overlap. These nested denominators are alternative, more conservative analytic frames; they do not create new eligibility decisions, meta-analytic datasets or causal estimates.

Auditability note: Some retained records had limited full-text auditability for selected descriptive fields despite the documented eligibility and verification workflow. The restricted-auditability frame (n = 292) makes this constraint explicit by excluding the 141 affected records. No claim of numerical invariance, ranking stability or unchanged evidence density is made here unless supported by a separately reported re-analysis; the sensitivity frames are provided to delimit the scope of inference and facilitate auditable follow-up analyses.

## Dense-cell direction-of-effect synthesis and focused risk-of-bias assessment

Four high-density exposure–AIRD cells were selected for additional targeted full-text re-coding: smoking/tobacco exposure × rheumatoid arthritis, air pollution/ambient exposure × rheumatoid arthritis, air pollution/ambient exposure × systemic lupus erythematosus, and occupational exposure × systemic sclerosis. Direction-of-effect coding was performed at the study-cell level using a SWiM-aligned framework. Final direction categories were positive/harmful, mixed, null/no clear association, inverse/protective, and not classifiable. Not-classifiable entries were retained in the audit trail but were not interpreted as directional votes.

A focused OHAT-style risk-of-bias assessment was performed for the same study-cell entries. Overall judgements were lower risk, some concerns, higher risk, unclear, or not assessed. Two reviewers independently performed targeted full-text verification, direction coding and focused risk-of-bias assessment. Disagreements were resolved by consensus adjudication. Inter-reviewer agreement was quantified using Cohen's κ for direction coding, for each focused risk-of-bias domain and for the overall focused risk-of-bias judgement. Direction coding agreement was κ = 0.95; focused RoB domain-level κ values ranged from 0.87 to 1.00, with overall focused RoB κ = 0.98 (Table S9). This dense-cell layer contextualises selected high-density cells and does not constitute a risk-of-bias assessment of the entire 433-study priority-coded map. Full record-level coding sheets, adjudication records and κ calculations are provided in the accompanying spreadsheets and Zenodo repository.

## Research-priority scoring framework

A consensus research-priority framework was applied across 64 exposure-family × AIRD cells defined by eight exposure families and eight AIRD groups. Three reviewers independently scored each cell across four 0–5 dimensions: scarcity-adjusted evidence gap, exposure prevalence or public-health relevance, biological plausibility, and AIRD burden or unmet need. Biological plausibility was operationalised through prespecified pathway domains, including mucosal or epithelial barrier hypotheses, oxidative stress, post-translational protein modification, epigenetic or endocrine immune modulation, innate/adaptive immune dysregulation, autoantibody formation and disease-specific immunopathology. The consensus score was the sum of median reviewer scores. Cells with a reviewer spread of at least 2 points in any dimension were adjudicated. Agreement was quantified using ICC(2,k) and the proportion of scores within ±1 point. Scores support research planning and do not represent association strength, effect size, evidence certainty, clinical recommendation or causality.

Each cell was scored against predefined 0–5 anchors for the four dimensions (Table S12). For the scarcity-adjusted dimension, an expected cell count was computed as a helper quantity and used to separate true cell-level gaps from globally under-studied diseases:

expected_n = (exposure-family row total × AIRD-column total) / total mapped exposure–AIRD entries (561); relative_gap_index = max(0, 1 − cell_n / expected_n) when expected_n > 0.

A guardrail for AIRD groups with low overall representation prevented zero cells from automatically receiving the highest scarcity scores unless an explicit external rationale was documented. Before the robustness analysis, current evidence volumes were reconciled against the final study-level AIRD assignments. Contextual non-AIRD components were not treated as separate Other/unspecified AIRD contributions; the corrected 8 × 8 matrix reproduced main Figure 3 and contained 17 zero-evidence cells. Post hoc robustness analyses omitted each scoring dimension in turn, halved the weight assigned to biological plausibility and doubled the weight assigned to scarcity. Spearman rank correlation and top-15 overlap were compared with the original four-dimension ranking.

## Post hoc immune-mechanism integration audit and dense-cell stratification

In response to peer review, each of the 433 included studies was assigned to the highest applicable level of a four-level hierarchy. Level 3 required direct measurement of a pathway-proximal intermediary or omics readout, such as cytokines, immune-cell or tissue pathways, oxidative or epigenetic markers, proteomics or transcriptomics. Level 2 required direct measurement of an immune, serologic, complement or inflammatory biomarker. Level 1 comprised immune-defined disease subtypes or genetic susceptibility/interaction without a measured intermediary. Level 0 denoted no integrated exposure-linked immune endpoint. Classification was based on the reported exposure–outcome analysis rather than on mechanistic statements in the background; use of an autoantibody solely for case definition was not sufficient for Level 2.

The audit used the frozen structured fields, titles, abstracts and available full-text or result-table verification notes. Records with pathway-proximal measurements, boundary status or limited auditability were prioritised for author verification. Results were summarised overall, by exposure family, by AIRD group and within the four dense cells. A boundary sensitivity analysis excluded the 11 authoritative boundary records. Dense-cell direction categories were additionally stratified by broad outcome type and exposure-assessment approach to clarify sources of mixed or positive/harmful classifications. The hierarchy measures the degree of immune integration and does not grade biomarker validity or mechanism certainty.

# **Supplementary Figures**

**Figure S1. Detailed AIRD distribution.**

Number of priority-coded studies per detailed AIRD category (n = 433). This figure expands the eight harmonised AIRD groups shown in main Fig. 2B into the more granular disease categories used during coding; a study reporting more than one AIRD is counted in each category, so counts can sum to more than 433 and percentages do not total 100%. Bars are coloured by disease family for readability, and family grouping does not alter the coded categories. Abbreviations: RA, rheumatoid arthritis; SLE, systemic lupus erythematosus; SSc, systemic sclerosis; pSS, primary Sjögren's syndrome; AS, ankylosing spondylitis; axSpA, axial spondyloarthritis; SpA, spondyloarthritis; PsA, psoriatic arthritis; IIM, idiopathic inflammatory myopathy; APS, antiphospholipid syndrome; AAV, ANCA-associated vasculitis; JIA/JRA, juvenile idiopathic/rheumatoid arthritis; CTD, connective tissue disease.

**Figure S2. Supplementary exposure-category × AIRD evidence matrix.**

Heatmap of the number of distinct priority-coded studies for every second-level exposure display category by AIRD group (n = 433); this is the granular numeric companion to the main evidence map (Fig. 3). Colour is square-root-scaled to keep small cells visible, and printed values are raw counts. A study may appear in several cells because exposure and disease categories are not mutually exclusive. Display-only merges were applied (PFAS → persistent organic pollutants; VOCs → organic solvents). Abbreviations: RA, rheumatoid arthritis; SLE, systemic lupus erythematosus; SSc, systemic sclerosis; pSS, primary Sjögren's syndrome; IIM, idiopathic inflammatory myopathy; PFAS, per- and polyfluoroalkyl substances; VOCs, volatile organic compounds.

Figure S3. Contextual author-keyword co-occurrence display.

Contextual co-occurrence display of author keywords from 308 of 433 priority-coded studies that reported keywords. Nodes are keywords occurring at least four times (n = 52), and edges denote co-occurrence in at least two studies. Node size is proportional to keyword frequency, node colour denotes modularity-based thematic clusters, and the layout is force-directed. For readability, only the most frequent or most connected keywords are labelled; frequency, degree, weighted degree and cluster membership for all 52 nodes are provided in Table S13. Keywords were lightly normalised for case, plurals and common synonyms. This display is descriptive and contextual; it was not used to determine evidence density, directionality, risk-of-bias judgements or research-priority scores, and it is not a citation-network analysis.

Figure S4. Top country/region distribution.

Number of priority-coded studies per reported country or region (n = 433). Multi-country studies are counted under each contributing country or region, so categories are not mutually exclusive. “Others” aggregates remaining individual countries that each contributed few studies, and “Not reported” denotes studies in which country or region was not stated. Bar colours denote world region and do not alter coding. This figure is the country-level companion to the choropleth map in main Fig. 4B and is descriptive only.

# **Supplementary Tables**

**Table S1. Full list of priority-coded studies (n = 433).**

Provided in full in the accompanying spreadsheet (sheet S1_study_list): record ID, authors, year, journal, DOI, study design, country/region, AIRD target and exposure domain as coded. The spreadsheet documents the frozen main-analysis set; the restricted-auditability sensitivity list is provided separately in the Zenodo audit materials.

Table S2. Exposure category × AIRD count matrix (distinct studies per cell).

| **Exposure category** | **RA** | **SLE** | **SSc** | **pSS** | **Spondyloarthritis** | **IIM** | **Vasculitis** | **Other/unspecified** | **Row total*** |
| --- | --- | --- | --- | --- | --- | --- | --- | --- | --- |
| Smoking / tobacco exposure | 136 | 35 | 8 | 9 | 32 | 2 | 5 | 15 | 215 |
| Occupational silica / mineral dust | 23 | 13 | 19 | 4 | 0 | 4 | 3 | 7 | 43 |
| Ambient air pollution or built environment | 20 | 13 | 3 | 2 | 2 | 1 | 0 | 0 | 41 |
| Air pollution | 14 | 13 | 4 | 5 | 5 | 4 | 0 | 12 | 26 |
| Metals | 9 | 2 | 2 | 0 | 1 | 0 | 0 | 0 | 14 |
| PFAS / persistent organic pollutants | 10 | 3 | 0 | 2 | 0 | 0 | 0 | 1 | 14 |
| Pesticides / farming / organic dust | 8 | 3 | 2 | 1 | 0 | 0 | 1 | 0 | 12 |
| Heavy metals / trace elements | 6 | 3 | 0 | 2 | 1 | 1 | 0 | 3 | 12 |
| Pesticides | 5 | 6 | 1 | 1 | 0 | 0 | 0 | 5 | 11 |
| Air pollution / particulate matter | 5 | 4 | 2 | 3 | 1 | 2 | 0 | 1 | 11 |
| VOCs / organic solvents | 4 | 2 | 3 | 1 | 0 | 1 | 0 | 1 | 10 |
| Occupational vapour/gas/dust/fume | 1 | 1 | 3 | 0 | 0 | 0 | 0 | 2 | 7 |
| Nitrogen dioxide | 2 | 1 | 1 | 2 | 1 | 1 | 0 | 1 | 5 |
| Polycyclic aromatic hydrocarbons | 4 | 0 | 0 | 0 | 0 | 0 | 0 | 1 | 4 |
| Bisphenol A | 1 | 2 | 1 | 1 | 0 | 0 | 0 | 0 | 4 |
| Diet-related chemicals / additives | 3 | 1 | 0 | 0 | 0 | 0 | 0 | 1 | 4 |
| Phthalates | 2 | 1 | 0 | 0 | 0 | 0 | 0 | 0 | 3 |
| Other eligible environmental agents | 2 | 0 | 0 | 0 | 0 | 0 | 0 | 0 | 2 |
| Diet-related chemicals / other eligible exposures | 1 | 0 | 0 | 0 | 1 | 0 | 0 | 0 | 2 |
| Carbon monoxide | 1 | 0 | 0 | 1 | 0 | 0 | 0 | 0 | 2 |
| Sulfur dioxide | 0 | 1 | 1 | 2 | 0 | 1 | 0 | 0 | 2 |
| Ambient environmental factors | 1 | 1 | 0 | 0 | 0 | 0 | 0 | 0 | 2 |
| Occupational exposure, unspecified | 0 | 0 | 0 | 0 | 0 | 1 | 0 | 1 | 1 |
| Ozone | 0 | 0 | 0 | 1 | 0 | 0 | 0 | 0 | 1 |
| Polybrominated biphenyls / organohalogens | 1 | 0 | 0 | 0 | 0 | 0 | 0 | 1 | 1 |
| Alcohol-related exposure (boundary-sensitive) | 0 | 0 | 0 | 0 | 1 | 0 | 0 | 0 | 1 |
| Diet-related chemicals / metals or minerals | 1 | 0 | 0 | 0 | 0 | 0 | 0 | 0 | 1 |
| Mixed environmental chemicals | 0 | 1 | 0 | 0 | 0 | 0 | 0 | 0 | 1 |
| Occupational dust | 1 | 0 | 0 | 0 | 0 | 0 | 0 | 0 | 1 |
| Occupational chemical disinfectants | 1 | 0 | 0 | 0 | 0 | 0 | 0 | 0 | 1 |
| Plasticizers / phthalate alternatives | 1 | 0 | 0 | 0 | 0 | 0 | 0 | 0 | 1 |
| *Row total = distinct studies addressing that exposure (a study may span several diseases, so row cells can sum to more than the row total). Display-only merges applied (PFAS→14; VOCs→10). |  |  |  |  |  |  |  |  |  |

A study may appear in several cells because categories are not mutually exclusive. Row total = distinct studies for that exposure. Display-only merges applied (PFAS -> 14; VOCs -> 10). Air-pollution second-level pollutant/display categories are non-mutually exclusive: one study measuring several pollutants or ambient exposure constructs may contribute to several second-level rows, so the air-pollution second-level row sum can exceed the first-level family total. Near-adjacent second-level labels are display categories rather than mutually exclusive mechanistic classes: for example, Pesticides / farming / organic dust denotes agricultural or organic-dust contexts with relevant exposure coding, whereas Pesticides denotes pesticide-agent records; Metals denotes the broader metal-exposure display domain, whereas Heavy metals / trace elements denotes specific element or biomarker-oriented records. Diet-related labels refer only to retained target chemical or boundary-coded exposure records; general diet or lifestyle-only variables were excluded.

**Table S3. Full-text exclusion reasons (PRISMA 2020; n = 2,196 excluded).**

| **Exclusion reason (predefined display label)** | **Studies, n** | **% of excluded** |
| --- | --- | --- |
| No eligible exposure–AIRD outcome link | 976 | 44.4% |
| Not an eligible environmental exposure | 936 | 42.6% |
| Non-original study or ineligible publication type | 150 | 6.8% |
| Not the target human AIRD population | 129 | 5.9% |
| Other full-text exclusion reasons | 4 | 0.2% |
| Duplicate or overlapping report | 1 | 0.0% |
| Total | 2196 |  |

Percentages are calculated using n = 2,196 full-text-assessed exclusions as denominator.

**Table S4. Display-term harmonisation dictionary.**

Provided in full in the accompanying spreadsheet (sheet S4_harmonization_dictionary): raw category, raw n, display category, harmonisation rule type and harmonisation-review flag for all variable groups.

Table S5. Evidence-sparse and unmapped exposure-family × AIRD combinations in the priority-coded set.

| **Exposure family** | **AIRD** | **n** | **Status** |
| --- | --- | --- | --- |
| A. No mapped study in the priority-coded set |  |  |  |
| Air pollution & ambient | Vasculitis | 0 | No mapped study in priority-coded set |
| Occupational | Spondyloarthritis | 0 | No mapped study in priority-coded set |
| Metals | Vasculitis | 0 | No mapped study in priority-coded set |
| Metals | Other / unspecified | 0 | No mapped study in priority-coded set |
| Pesticides | Spondyloarthritis | 0 | No mapped study in priority-coded set |
| Pesticides | IIM | 0 | No mapped study in priority-coded set |
| Solvents, VOCs & POPs | Spondyloarthritis | 0 | No mapped study in priority-coded set |
| Solvents, VOCs & POPs | Vasculitis | 0 | No mapped study in priority-coded set |
| Endocrine disruptors & additives | Spondyloarthritis | 0 | No mapped study in priority-coded set |
| Endocrine disruptors & additives | IIM | 0 | No mapped study in priority-coded set |
| Endocrine disruptors & additives | Vasculitis | 0 | No mapped study in priority-coded set |
| Endocrine disruptors & additives | Other / unspecified | 0 | No mapped study in priority-coded set |
| Diet-related chemicals & other eligible exposures | SSc | 0 | No mapped study in priority-coded set |
| Diet-related chemicals & other eligible exposures | pSS | 0 | No mapped study in priority-coded set |
| Diet-related chemicals & other eligible exposures | IIM | 0 | No mapped study in priority-coded set |
| Diet-related chemicals & other eligible exposures | Vasculitis | 0 | No mapped study in priority-coded set |
| Diet-related chemicals & other eligible exposures | Other / unspecified | 0 | No mapped study in priority-coded set |
| B. Very limited mapped evidence (1-3 studies) |  |  |  |
| Diet-related chemicals & other eligible exposures | RA | 2 | Very limited |
| Diet-related chemicals & other eligible exposures | Spondyloarthritis | 2 | Very limited |
| Diet-related chemicals & other eligible exposures | SLE | 1 | Very limited |
| Endocrine disruptors & additives | SSc | 1 | Very limited |
| Endocrine disruptors & additives | pSS | 1 | Very limited |
| Metals | SSc | 2 | Very limited |
| Metals | pSS | 2 | Very limited |
| Metals | Spondyloarthritis | 2 | Very limited |
| Metals | IIM | 1 | Very limited |
| Occupational | Vasculitis | 3 | Very limited |
| Pesticides | SSc | 3 | Very limited |
| Pesticides | pSS | 2 | Very limited |
| Pesticides | Vasculitis | 1 | Very limited |
| Smoking & tobacco | IIM | 2 | Very limited |
| Solvents, VOCs & POPs | SSc | 3 | Very limited |
| Solvents, VOCs & POPs | pSS | 3 | Very limited |
| Solvents, VOCs & POPs | Other / unspecified | 2 | Very limited |
| Solvents, VOCs & POPs | IIM | 1 | Very limited |

Derived from the exposure-family × AIRD evidence map (main Figure 3). 'No mapped study in priority-coded set' = no retained study in the priority-coded map; 'Very limited mapped evidence' = 1–3 studies. These labels do not imply absence of association or absence of any study outside the priority-coded map.

**Table S6. Exposure-family research volume and recency.**

| **Exposure family** | **Studies n** | **2021-2025 n** | **Recent share** | **Profile** |
| --- | --- | --- | --- | --- |
| Smoking & tobacco | 215 | 54 | 25.1% | Established (high volume) |
| Air pollution & ambient | 83 | 56 | 67.5% | Mixed / steady |
| Occupational | 53 | 25 | 47.2% | Established (high volume) |
| Metals | 27 | 14 | 51.9% | Mixed / steady |
| Solvents, VOCs & POPs | 25 | 18 | 72.0% | Emerging (recent-weighted) |
| Pesticides | 23 | 8 | 34.8% | Mixed / steady |
| Endocrine disruptors & additives | 12 | 12 | 100.0% | Emerging (recent-weighted) |
| Diet-related chemicals & other eligible exposures | 5 | 5 | 100.0% | Emerging (recent-weighted) |

Recent share = studies published in 2021-2025 as a fraction of that family's studies.

**Table S7. Source journals within the priority-coded map (top 15 of 139; contextual descriptor).**

| **Rank** | **Journal** | **n** | **% of 433** |
| --- | --- | --- | --- |
| 1 | Annals Of The Rheumatic Diseases | 32 | 7.4% |
| 2 | Arthritis Care And Research | 22 | 5.1% |
| 3 | Rheumatology International | 16 | 3.7% |
| 4 | Arthritis And Rheumatology | 15 | 3.5% |
| 5 | Rheumatology (United Kingdom) | 14 | 3.2% |
| 6 | Arthritis And Rheumatism | 13 | 3.0% |
| 7 | Journal Of Rheumatology | 13 | 3.0% |
| 8 | Lupus | 12 | 2.8% |
| 9 | Clinical Rheumatology | 12 | 2.8% |
| 10 | Arthritis Research And Therapy | 12 | 2.8% |
| 11 | Rmd Open | 10 | 2.3% |
| 12 | Environmental Health Perspectives | 9 | 2.1% |
| 13 | Environmental Research | 9 | 2.1% |
| 14 | International Journal Of Environmental Research And Public Health | 9 | 2.1% |
| 15 | Science Of The Total Environment | 8 | 1.8% |

Percentages use n = 433 as denominator. This table is a contextual description of the journals represented in the priority-coded map; it was not used to determine evidence density, directionality, risk-of-bias judgements or research priorities.

## Table S8. Summary of dense-cell direction-of-effect synthesis and focused risk-of-bias assessment.

| **Exposure family × AIRD cell** | **Mapped cell n** | **Direction-vote entries** | **Positive/harmful** | **Mixed** | **Null/no clear** | **Not classifiable** | **Higher-risk share among direction-vote entries** |
| --- | --- | --- | --- | --- | --- | --- | --- |
| Smoking/tobacco × RA | 136 | 125 | 37 | 77 | 11 | 11 | 3% |
| Air pollution/ambient × RA | 43 | 34 | 21 | 10 | 3 | 9 | 26% |
| Air pollution/ambient × SLE | 30 | 21 | 16 | 4 | 1 | 9 | 57% |
| Occupational exposure × SSc | 22 | 19 | 17 | 2 | 0 | 3 | 79% |
| Total | 231 | 199 | 91 | 93 | 15 | 32 | 40 higher-risk entries overall |

RA, rheumatoid arthritis; SLE, systemic lupus erythematosus; SSc, systemic sclerosis. Not-classifiable entries were retained in the audit trail but were not interpreted as directional votes. Higher-risk share refers to the proportion of direction-vote entries judged as higher risk of bias. The accompanying record-level dense-cell spreadsheet reports the exposure metric, outcome definition, selected adjusted model, effect estimate and 95% confidence interval where extractable, result-table or page location, exact supporting result text, final direction, focused risk-of-bias judgement and adjudication record for every study-cell entry.

## Table S9. Inter-reviewer agreement and consensus adjudication summary for dense-cell synthesis.

| **Item** | **Value** | **Interpretive note** |
| --- | --- | --- |
| Study-cell entries in final dense-cell audit dataset | 231 | Four high-density exposure–AIRD cells. |
| Direction-vote entries | 199 | Not-classifiable entries excluded from directional interpretation. |
| Not-classifiable entries | 32 | Retained in audit trail. |
| Direction disagreements before adjudication | 7 | Resolved by consensus. |
| Focused RoB disagreements before adjudication | 2 | Resolved by consensus. |
| Direction Cohen’s κ | 0.95 | High inter-reviewer agreement; rounded to two decimals in text. |
| Focused RoB overall Cohen's κ | 0.98 | Overall focused RoB agreement; domain-level κ values are reported separately. |
| Final direction distribution | mixed 93; positive/harmful 91; null/no clear 15; not classifiable 32 | No pooled effect estimates were calculated. |
| Final focused RoB distribution | some concerns 153; higher risk 40; not assessed 31; lower risk 6; unclear 1 | Focused RoB layer was not a full 433-study RoB assessment. |
| Focused RoB domain-level Cohen's κ range | 0.87-1.00 | Domain-level values provide the gradient behind the high overall agreement. |
| Priority scoring ICC(2,k) range | 0.91-0.94 | Three-reviewer consensus scoring across the four priority dimensions. |
| Priority scoring within ±1 point | 97-100% | Proportion of reviewer scores within ±1 point across scoring dimensions. |

## Table S10. Research-priority scoring framework across 64 exposure–AIRD combinations.

| **Dimension** | **Score range** | **Meaning of higher score** | **Interpretive caution** |
| --- | --- | --- | --- |
| Scarcity-adjusted evidence gap | 0–5 | Greater evidence scarcity or underdevelopment after considering the evidence map. | Does not imply absence of effect. |
| Exposure prevalence / public-health relevance | 0–5 | More widespread, modifiable, occupationally important or policy-relevant exposure. | Does not imply stronger association. |
| Biological plausibility | 0–5 | Stronger mechanistic rationale linking the exposure family and AIRD group. | Does not prove causality. |
| AIRD burden / unmet need | 0–5 | Greater clinical burden, underrepresentation or unmet environmental-research need. | Does not grade evidence certainty. |
| Consensus priority score | 0–20 | Sum of median reviewer scores across the four dimensions. | Used for research planning only. |
| Consensus and adjudication | Median score; ≥2-point spread adjudicated | Consensus medians reduced sensitivity to outlying single-reviewer ratings. | Reviewer agreement was high, but the framework remains judgement-based and hypothesis-generating. |
| Scarcity adjustment | 0–5 | Observed cell count interpreted against expected evidence volume within the 64-cell exposure-family × AIRD matrix. | Distinguishes true evidence voids from disease-wide or exposure-wide underrepresentation. |

## Table S11. Top 15 research-priority combinations and scoring components.

*A. Cell counts and evidence status.*

| **Rank** | **Exposure family × AIRD combination** | **Cell n** | **Evidence status** | **Consensus score** |
| --- | --- | --- | --- | --- |
| 1 | Smoking & tobacco × SSc | 8 | Limited | 16 |
| 2 | Occupational × Spondyloarthritis | 0 | No mapped study in priority-coded set | 15 |
| 3 | Smoking & tobacco × IIM | 2 | Very limited | 15 |
| 4 | Air pollution & ambient × Vasculitis | 0 | No mapped study in priority-coded set | 15 |
| 5 | Diet-related chemicals & other eligible exposures × SSc | 0 | No mapped study in priority-coded set | 14 |
| 6 | Solvents, VOCs & POPs × SSc | 3 | Very limited | 14 |
| 7 | Air pollution & ambient × SLE | 30 | Moderate | 14 |
| 8 | Smoking & tobacco × SLE | 35 | Higher-volume | 14 |
| 9 | Air pollution & ambient × SSc | 9 | Limited | 14 |
| 10 | Occupational × SSc | 22 | Moderate | 14 |
| 11 | Pesticides × Spondyloarthritis | 0 | No mapped study in priority-coded set | 13 |
| 12 | Solvents, VOCs & POPs × Spondyloarthritis | 0 | No mapped study in priority-coded set | 13 |
| 13 | Diet-related chemicals & other eligible exposures × pSS | 0 | No mapped study in priority-coded set | 13 |
| 14 | Diet-related chemicals & other eligible exposures × RA | 2 | Very limited | 13 |
| 15 | Diet-related chemicals & other eligible exposures × IIM | 0 | No mapped study in priority-coded set | 13 |

*B. Scarcity adjustment and component scores.*

| **Rank** | **Expected n** | **Relative gap index** | **Median component scores (gap/exposure/plausibility/burden)** | **Consensus score** |
| --- | --- | --- | --- | --- |
| 1 | 18.4 | 0.57 | 2/5/4/5 | 16 |
| 2 | 4.0 | 1.00 | 5/4/3/3 | 15 |
| 3 | 5.4 | 0.63 | 3/5/3/4 | 15 |
| 4 | 1.3 | 1.00 | 2/5/4/4 | 15 |
| 5 | 0.4 | 1.00 | 2/4/3/5 | 14 |
| 6 | 2.1 | 0.00 | 2/3/4/5 | 14 |
| 7 | 14.9 | 0.00 | 1/5/4/4 | 14 |
| 8 | 38.7 | 0.10 | 1/5/4/4 | 14 |
| 9 | 7.1 | 0.00 | 1/5/3/5 | 14 |
| 10 | 4.5 | 0.00 | 1/4/4/5 | 14 |
| 11 | 1.7 | 1.00 | 4/3/3/3 | 13 |
| 12 | 1.9 | 1.00 | 4/3/3/3 | 13 |
| 13 | 0.3 | 1.00 | 2/4/4/3 | 13 |
| 14 | 2.3 | 0.12 | 2/4/4/3 | 13 |
| 15 | 0.1 | 1.00 | 2/4/3/4 | 13 |

Priority scores are intended to support future research planning. They do not represent effect sizes, evidence certainty, clinical recommendations or causal inference. Expected n and the relative-gap index were helper quantities used to inform the scarcity dimension rather than automatic scores. Post hoc alternative-weight and leave-one-dimension-out analyses are reported in Table S16. Corrected evidence-volume values were used for the revised display; no evidence-density correction changed the direction-of-effect synthesis or the scores of the principal non-Other priority cells.

**Table S13. Node metrics for the contextual author-keyword co-occurrence display.**

Contextual display of author keywords from 308 of 433 priority-coded studies reporting keywords. Nodes = keywords occurring ≥4 times; edges = co-occurrence in the same study (≥2). Network: 52 nodes, 144 edges, 5 modularity-based clusters (modularity = 0.25). Frequency = number of studies in which the keyword appears; degree = number of co-occurring keywords; weighted degree = summed co-occurrence weight. Keywords were lightly normalised (case, plurals, common synonyms).

| **Keyword** | **n** | **Deg.** | **Wtd.** **degree** | **Cluster** | **Cluster label** **(lead keyword)** |
| --- | --- | --- | --- | --- | --- |
| rheumatoid arthritis | 122 | 32 | 189 | 1 | C1: rheumatoid arthritis |
| smoking | 116 | 29 | 213 | 1 | C1: rheumatoid arthritis |
| epidemiology | 29 | 9 | 54 | 1 | C1: rheumatoid arthritis |
| risk factors | 24 | 11 | 58 | 1 | C1: rheumatoid arthritis |
| nhanes | 16 | 4 | 19 | 1 | C1: rheumatoid arthritis |
| autoantibodies | 9 | 2 | 11 | 1 | C1: rheumatoid arthritis |
| shared epitope | 7 | 2 | 14 | 1 | C1: rheumatoid arthritis |
| inflammation | 6 | 3 | 9 | 1 | C1: rheumatoid arthritis |
| interaction | 5 | 3 | 10 | 1 | C1: rheumatoid arthritis |
| heavy metals | 5 | 2 | 5 | 1 | C1: rheumatoid arthritis |
| spondyloarthritis | 5 | 1 | 2 | 1 | C1: rheumatoid arthritis |
| rheumatology | 5 | 1 | 2 | 1 | C1: rheumatoid arthritis |
| sjogren's syndrome | 5 | 1 | 2 | 1 | C1: rheumatoid arthritis |
| environmental factors | 5 | 1 | 2 | 1 | C1: rheumatoid arthritis |
| rheumatoid factor | 4 | 3 | 9 | 1 | C1: rheumatoid arthritis |
| polycyclic aromatic hydrocarbons | 4 | 2 | 4 | 1 | C1: rheumatoid arthritis |
| alcohol consumption | 4 | 1 | 3 | 1 | C1: rheumatoid arthritis |
| psoriasis | 4 | 1 | 2 | 1 | C1: rheumatoid arthritis |
| systemic lupus erythematosus | 48 | 16 | 68 | 2 | C2: systemic lupus erythematosus |
| air pollution | 46 | 15 | 70 | 2 | C2: systemic lupus erythematosus |
| ankylosing spondylitis | 20 | 8 | 34 | 2 | C2: systemic lupus erythematosus |
| disease activity | 19 | 9 | 40 | 2 | C2: systemic lupus erythematosus |
| particulate matter | 13 | 9 | 31 | 2 | C2: systemic lupus erythematosus |
| lupus nephritis | 6 | 2 | 6 | 2 | C2: systemic lupus erythematosus |
| axial spondyloarthritis | 5 | 3 | 10 | 2 | C2: systemic lupus erythematosus |
| nitrogen dioxide | 5 | 2 | 6 | 2 | C2: systemic lupus erythematosus |
| quality of life | 4 | 4 | 10 | 2 | C2: systemic lupus erythematosus |
| mendelian randomization | 4 | 3 | 7 | 2 | C2: systemic lupus erythematosus |
| psoriatic arthritis | 4 | 2 | 5 | 2 | C2: systemic lupus erythematosus |
| pm10 | 4 | 2 | 6 | 2 | C2: systemic lupus erythematosus |
| fine particulate matter | 4 | 2 | 6 | 2 | C2: systemic lupus erythematosus |
| cohort study | 4 | 2 | 6 | 2 | C2: systemic lupus erythematosus |
| dermatomyositis | 4 | 1 | 2 | 2 | C2: systemic lupus erythematosus |
| environmental exposure | 4 | 1 | 2 | 2 | C2: systemic lupus erythematosus |
| systemic sclerosis | 25 | 12 | 36 | 3 | C3: systemic sclerosis |
| autoimmune diseases | 21 | 12 | 29 | 3 | C3: systemic sclerosis |
| silica | 15 | 8 | 28 | 3 | C3: systemic sclerosis |
| occupational exposure | 11 | 5 | 14 | 3 | C3: systemic sclerosis |
| interstitial lung disease | 8 | 4 | 12 | 3 | C3: systemic sclerosis |
| silicosis | 7 | 3 | 8 | 3 | C3: systemic sclerosis |
| environment | 6 | 6 | 14 | 3 | C3: systemic sclerosis |
| solvents | 6 | 5 | 12 | 3 | C3: systemic sclerosis |
| occupation | 6 | 5 | 12 | 3 | C3: systemic sclerosis |
| pesticides | 6 | 3 | 7 | 3 | C3: systemic sclerosis |
| sjögren’s syndrome | 4 | 2 | 4 | 3 | C3: systemic sclerosis |
| arthritis | 18 | 7 | 29 | 4 | C4: arthritis |
| autoimmunity | 15 | 7 | 24 | 4 | C4: arthritis |
| rheumatoid | 7 | 5 | 19 | 4 | C4: arthritis |
| anti-citrullinated protein antibodies | 4 | 4 | 10 | 4 | C4: arthritis |
| systemic | 7 | 4 | 12 | 5 | C5: systemic |
| polymorphism | 5 | 4 | 10 | 5 | C5: systemic |
| lupus erythematosus | 5 | 3 | 9 | 5 | C5: systemic |

Note: Frequency is the number of priority-coded studies in which the normalised author keyword appears. Degree is the number of distinct co-occurring keyword partners, and weighted degree is the summed co-occurrence weight. Cluster labels are descriptive lead-keyword labels derived from the top terms in each modularity-based cluster. This table supports interpretation of Fig. S3 and is a contextual display, not an analysis of exposure–disease associations, causal evidence or citation structure.

**Table S12. Research-priority scoring rubric (0–5 anchors for each of the four dimensions).**

| **Dimension** | **0** | **1–2** | **3** | **4–5** |
| --- | --- | --- | --- | --- |
| Scarcity-adjusted evidence gap | Not scoreable; cell should be split, merged or excluded. | Not a priority gap: cell adequately represented (cell_n >10 or relative_gap_index <0.50), or sparse mainly because the AIRD/exposure family is globally low-volume. | Moderate relative gap or uncertain blank (cell_n 1–3 with relative_gap_index 0.50–0.75, or zero cell in an AIRD with 6–11 total studies). | Strong relative gap to true blank in an otherwise-studied AIRD (cell_n 0–1, AIRD_total_n ≥12–20, expected_n ≥2–3, or relative_gap_index ≥0.75). |
| Exposure prevalence / public-health relevance | Not scoreable or no plausible public-health lever. | Rare, transient or poorly measured exposure with limited scalable intervention. | Measurable, relevant exposure with narrower reach (≈1–9% population or a defined high-risk subgroup) and some actionability. | Common to ubiquitous exposure (≥10% population reach or a large occupational/community burden) with a credible policy, occupational, clinical or environmental intervention lever. |
| Biological plausibility | No or weak mechanistic basis. | Indirect or limited mechanistic rationale. | Moderate mechanistic and/or epidemiological rationale. | Strong, established mechanistic and epidemiological rationale for the exposure–AIRD relationship. |
| AIRD burden / unmet need | Not scoreable. | Well studied or low unmet need. | Moderate disease burden or unmet need. | High disease burden combined with under-representation or high unmet need. |

Scores are hypothesis-generating research priorities and do not represent effect size, evidence certainty or causal association. The complete per-cell scores, rationales and adjudication records are provided in the deposited files.

**Table S14. Four-level immune-mechanism integration audit in the overall map and dense cells.**

| **Population/cell** | **Total n** | **Level 3** | **Level 2** | **Level 1** | **Level 0** |
| --- | --- | --- | --- | --- | --- |
| Overall map | 433 | 25 | 79 | 63 | 266 |
| Smoking/tobacco × RA | 136 | 6 | 40 | 39 | 51 |
| Air pollution × RA | 43 | 1 | 3 | 3 | 36 |
| Air pollution × SLE | 30 | 3 | 3 | 0 | 24 |
| Occupational × SSc | 22 | 1 | 5 | 1 | 15 |

Levels are mutually exclusive and assigned at study level. Level 3 = pathway-proximal intermediary/omics; Level 2 = direct immune/serologic, complement or inflammatory biomarker; Level 1 = immune-defined subtype or genetic susceptibility/interaction; Level 0 = no integrated exposure-linked immune endpoint.

Table S15A. Immune-mechanism integration by exposure family (harmonised exposure-family labels).

| **Exposure family** | **Total** | **L3** | **L2** | **L1** | **L0** | **L2–3 %** | **L1–3 %** |
| --- | --- | --- | --- | --- | --- | --- | --- |
| Smoking & tobacco | 215 | 12 | 54 | 46 | 103 | 30.7% | 52.1% |
| Air pollution & ambient | 83 | 4 | 6 | 3 | 70 | 12.0% | 15.7% |
| Occupational | 53 | 2 | 10 | 10 | 31 | 22.6% | 41.5% |
| Metals | 27 | 4 | 2 | 1 | 20 | 22.2% | 25.9% |
| Pesticides | 23 | 1 | 2 | 1 | 19 | 13.0% | 17.4% |
| Solvents, VOCs & POPs | 25 | 1 | 6 | 1 | 17 | 28.0% | 32.0% |
| Endocrine disruptors & additives | 12 | 1 | 0 | 1 | 10 | 8.3% | 16.7% |
| Diet-related chemicals & other eligible environmental agents | 5 | 1 | 1 | 1 | 2 | 40.0% | 60.0% |

**Table S15B. Immune-mechanism integration by AIRD group.**

| **AIRD group** | **Total** | **L3** | **L2** | **L1** | **L0** | **L2–3 %** | **L1–3 %** |
| --- | --- | --- | --- | --- | --- | --- | --- |
| RA | 254 | 9 | 52 | 53 | 140 | 24.0% | 44.9% |
| SLE | 101 | 11 | 12 | 9 | 69 | 22.8% | 31.7% |
| SSc | 48 | 1 | 10 | 2 | 35 | 22.9% | 27.1% |
| pSS | 32 | 3 | 4 | 2 | 23 | 21.9% | 28.1% |
| Spondyloarthritis | 42 | 3 | 6 | 0 | 33 | 21.4% | 21.4% |
| IIM | 14 | 0 | 4 | 2 | 8 | 28.6% | 42.9% |
| Vasculitis | 9 | 0 | 0 | 2 | 7 | 0.0% | 22.2% |
| Other / unspecified | 37 | 2 | 10 | 1 | 24 | 32.4% | 35.1% |

Disease and exposure groupings are non-mutually exclusive across studies, but each study receives one immune-integration level. Percentages use the corresponding group total.

**Table S16. Robustness of the 64-cell research-priority ranking.**

| **Alternative scheme** | **Spearman ρ** | **Top-15 overlap** | **Smoking × SSc rank** | **Smoking × SSc score** |
| --- | --- | --- | --- | --- |
| Omit biological plausibility | 0.956 | 12 | 2 | 15 |
| Omit scarcity | 0.871 | 13 | 1 | 13 |
| Omit public-health relevance | 0.855 | 10 | 2 | 13 |
| Omit AIRD burden/unmet need | 0.905 | 9 | 3 | 12 |
| Biological plausibility half-weight | 0.991 | 12 | 1 | 14.5 |
| Scarcity double-weight | 0.955 | 10 | 2.5 | 18 |

Correlations and top-15 overlaps are compared with the original four-dimension ranking. Average ranks reflect ties. Smoking/tobacco × systemic sclerosis remained within ranks 1–3 across all alternative schemes.

**Table S17. Direction categories in dense cells stratified by broad outcome type.**

| **Dense cell** | **Outcome stratum** | **Vote n** | **Positive/harmful** | **Mixed** | **Null/no clear** |
| --- | --- | --- | --- | --- | --- |
| Smoking/tobacco × RA | Disease activity/phenotype or clinical course | 26 | 4 | 20 | 2 |
| Smoking/tobacco × RA | Disease occurrence/prevalence | 37 | 14 | 20 | 3 |
| Smoking/tobacco × RA | Immune/serologic or molecular endpoint | 62 | 19 | 37 | 6 |
| Air pollution × RA | Disease activity/phenotype or clinical course | 14 | 7 | 5 | 2 |
| Air pollution × RA | Disease occurrence/prevalence | 15 | 11 | 3 | 1 |
| Air pollution × RA | Immune/serologic or molecular endpoint | 5 | 3 | 2 | 0 |
| Air pollution × SLE | Disease activity/phenotype or clinical course | 15 | 11 | 3 | 1 |
| Air pollution × SLE | Disease occurrence/prevalence | 6 | 5 | 1 | 0 |
| Occupational × SSc | Disease activity/phenotype or clinical course | 12 | 11 | 1 | 0 |
| Occupational × SSc | Disease occurrence/prevalence | 5 | 4 | 1 | 0 |
| Occupational × SSc | Immune/serologic or molecular endpoint | 2 | 2 | 0 | 0 |

Counts include the 199 classifiable study-cell entries. Broad outcome strata were assigned from the frozen outcome-domain and outcome-measure fields to describe sources of directional heterogeneity; they are not pooled effect strata.

Table S18. Study-cell-level details for the four dense exposure–AIRD cells (provided as a separate XLSX supplementary table).

Table S18 lists the exposure assessment, outcome definition, selected adjustment set/model, effect estimate and confidence interval where extractable, direction judgement, focused risk-of-bias judgement, result location and adjudication information for all 231 study-cell entries.

Full record-level coding sheets, dense-cell result and adjudication records, Table S18, the signed four-level mechanism-integration audit, corrected 64-cell matrix, priority-ranking robustness analyses and restricted-auditability sensitivity materials are provided in the accompanying spreadsheet files and will be included in the updated Zenodo repository version accompanying the revision. No copyrighted full-text articles are redistributed.
